# Synthesis and pyroelectric response of disperse red 1 functionalized silicones: cyclic monomer, homopolymer, and block copolymer derivatives

**DOI:** 10.1039/d6mh00410e

**Published:** 2026-05-04

**Authors:** Malte Sebastian Beccard, Thulasinath Raman Venkatesan, Francesco Taddei, Laura Baraldi, Raffaele Mezzenga, Frank A. Nüesch, Dorina M. Opris

**Affiliations:** a Laboratory of Functional Polymers, Empa, Swiss Federal Laboratories for Materials Science and Technology (EMPA) 8600 Dübendorf Switzerland Dorina.opris@empa.ch; b Department of Materials, ETH Zürich 8092 Zurich Switzerland; c Mechanical System Engineering, Swiss Federal Laboratories for Materials Science and Technology – Empa Dübendorf Switzerland; d Department of Health Sciences and Technology, ETH Zürich, Laboratory of Food and Soft Materials 8092 Zürich Switzerland; e Institute of Chemical Sciences and Engineering, Ecole Polytechnique Federale de Lausanne, EPFL, Station 6 CH-1015 Lausanne Switzerland

## Abstract

Pyroelectric materials enable the direct conversion of thermal fluctuations into electrical energy, offering a promising approach to waste heat recovery. While pyroelectric polymers are highly valued for their scalable synthesis, mechanical flexibility, and tunable properties, the field is currently dominated by poly(vinylidene fluoride) (PVDF)-based materials, which present environmental and processing challenges. To develop fluorine-free alternatives and elucidate the influence of molecular architecture on thermal-to-electrical conversion, we synthesized a series of siloxane-based materials functionalized with Disperse Red 1 (DR1) moieties, including a cyclic siloxane monomer, a homopolysiloxane, and a block copolysiloxane. Differential scanning calorimetry confirms the semicrystalline nature of these siloxanes, with glass transitions (*T*_g_) near room temperature and melting temperatures of about 80 °C. Notably, even unpoled samples exhibit a measurable pyroelectric response at elevated temperatures. The pyroelectric response at low temperatures is significantly enhanced by poling the crystalline domains in an electric field above the melting transitions (*T*_m_). Among the synthesized materials, the homopolymer exhibited the highest pyroelectric response (0.66 µC m^−2^ K^−1^ at 60 °C). While this value is significantly lower than the typical values for PVDF (>20 µC m^−2^ K^−1^), it should be noted that the processing and poling steps differ substantially. Under similar conditions, the PVDF value was only twice that of the homopolymer. Even more interesting, in an unpoled sample, the homopolymer shows a response similar to that of the poled sample, while PVDF shows almost no response. The superior response for the unpoled sample is attributed to the synergistic effects of DR1 self-ordering and secondary pyroelectricity—the strain-induced changes in dipole density resulting from thermal expansion. These findings provide a framework for designing high-performance, silicone-based pyroelectric transducers through precise structural control.

## Abstract 2

Self-assembled H-aggregates in Disperse Red-functionalized polysiloxane yield spontaneous pyroelectricity. Remarkably, this material achieves pyroelectric performance comparable to that of PVDF processed under similar conditions.

## Graphical abstract

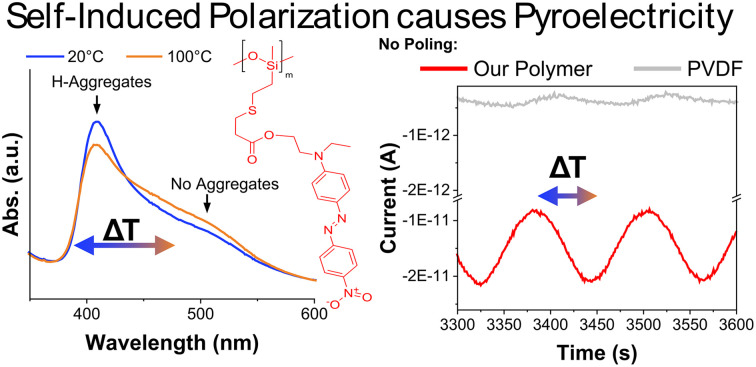

**New concepts**

This work introduces fluorine-free, polysiloxane-based materials for pyroelectricity, shifting from the conventional semi-crystalline fluoropolymer paradigm. We demonstrate that pyroelectricity can be achieved by exploiting H-aggregate formation within a highly functionalized polysiloxane matrix. This approach shifts the design focus from the forced alignment of ferroelectric crystalline domains to the architectural control of chromophore stacking, providing a fluorine-free alternative to conventional fluorinated polymers. The distinguishing feature of this study is the identification and exploitation of stable polarization in unpoled samples, which persists even at elevated temperatures. In contrast to many other materials, which undergo dipole randomisation once the matrix becomes mobile, resulting in a loss of pyroelectric response, it is demonstrated that H-aggregates persist, thereby enabling a measurable response without the necessary post-processing external high electric-field treatment. This work demonstrates that molecular architecture, rather than high voltage poling, can lead to pyroelectricity in soft matter. By showing how structural motifs (cyclic, homo-, and block-copolymers) govern aggregate stability, we provide a toolkit for designing “self-polarised” materials that could serve as an alternative to current fluorinated polymers.

## Introduction

Electricity is an indispensable resource for technological innovation, fuelling the development of the Internet of Things (IoT). By powering a vast network of interconnected devices, it has fundamentally reshaped both industrial processes and daily life.^1,2^ However, energy resources on our planet remain limited, underscoring the need to optimize energy consumption, minimize waste, and generate power locally where it is needed.^2,3^ One promising strategy to address these challenges is using pyroelectric materials, which convert thermal fluctuations into electrical energy.^2–5^ Pyroelectric materials have a wide range of applications, including sensors such as those to detect fire, light, or gas sensors,^6^ as well as thermal imaging.^6^ They are also used in pyro-electrochemical processes, such as the generation of hydrogen, as well as in sterilization and disinfection.^4^ Pyroelectricity is found in polar materials that exhibit spontaneous or induced electric polarization. This polarization can be modulated by temperature variations, producing transient voltage and charge. As the temperature increases, the dipoles in the dielectric material fluctuate more, reducing the polarization strength. Conversely, as the temperature decreases, dipole fluctuations decrease and polarization increases. This change in polarization can be used to generate electricity.^2–4,7,8^ It is important to note that dipole fluctuations are not the only factors contributing to changes in the performance of a pyroelectric material. The coefficient of thermal expansion also contributes to this process by inducing thermal strain and volume expansion. These result in a reduced dipole density, which, in turn, causes a secondary pyroelectric effect.^3,7,9,10^ In many cases, the primary pyroelectric effect is significantly larger than the secondary one.^6,11^ However, in certain materials, such as tourmaline or bone, the secondary pyroelectric effect can contribute most to the total pyroelectric response.^12^

A variety of pyroelectric materials exists,^2^ including inorganic single crystals,^13^ ceramics,^14^ inorganic thin films,^15^ or polymers and composites.^16^ The pyroelectric effect was demonstrated not only in semicrystalline and liquid crystalline polymers, but also in amorphous polymers.^17^ To qualify as a suitable pyroelectric material, a polymer must contain molecular dipoles, which can be polarized, and these dipoles must maintain the polarization for an extended time, even when exposed to fluctuations in temperature.^17^ Additionally, polymers offer several advantages, such as being lightweight, flexible, chemically resistant, biocompatible, and easy to process.^4,18^ The most explored pyroelectric polymer is poly(vinylidene difluoride) (PVDF) (pyroelectric coefficient, *p*-coefficient ≥ 30 µC m^−2^ K^−1^),^19,20^ which exhibits a low glass transition temperature (*T*_g_) of −35 °C and multiple crystalline phases at elevated temperatures.^21,22^

However, PVDF is synthesized from fluorinated monomers, which form long-lasting, toxic pollutants.^23^ Other polymers that have been investigated for their pyroelectricity are, for example, polyamides,^24^ polyureas,^25^ and polythioureas,^26^ however, their pyroelectric response is rather low. The high backbone flexibility and ease of functionalization make polysiloxanes an ideal platform for developing polar polymers. By strategically selecting polar side groups, the electrical properties of these siloxanes can be enhanced while precisely tuning the *T*_g_ to suit specific applications.^27–29^ Despite their versatile properties, polysiloxanes have remained largely unexplored as pyroelectric materials. Early investigations, however, have demonstrated that Langmuir–Blodgett films composed of specific siloxane copolymers can achieve significant pyroelectric coefficients, suggesting that structured silicone architectures hold untapped potential for thermal energy harvesting.^30^ Recent studies have explored PDMS-based composites containing polar amorphous fillers. Poling these fillers above their *T*_g_ within the flexible siloxane matrix enables the development of stable remanent polarization, yielding functional piezoelectric and pyroelectric properties.^16,31,32^ Another interesting approach was reported by Mauzac and coworkers, who synthesized liquid-crystalline polysiloxanes.^33^ Depending on the composition, the crosslinked polymers showed *p*-coefficient values up to 140 pC cm^−2^ K^−1^. Not only the chemical structure but also the material's assembly in the polymer greatly influences its dielectric properties.^34^ The dielectric properties of a polymer matrix can vary depending on the interaction between the polymer and filler, meaning that the polymer's dielectric response can differ in the bulk and at the filler interface.^35^

In this study, we synthesized a series of siloxane architectures—comprising a cyclotetrasiloxane, a homopolysiloxane, and a block copolysiloxane, each functionalized with Disperse Red 1 (DR1) to investigate the correlation between macromolecular structure and pyroelectric performance. DR1 was selected as the active dipolar moiety due to its substantial dipole moment, which will increase the polarization after dielectric poling.^32,36^ The cyclotetrasiloxane was also selected because it is commonly present in polysiloxanes at about 15 wt% as a contaminant and could thus significantly affect the pyroelectric response.^37^ The structural diversity of our strategy enabled us to observe the impact of phase separation and interphase effects on pyroelectricity.

## Experimental

The structure of the three different siloxanes (see synthesis below) was confirmed using ^1^H, ^13^C, and ^29^Si nuclear magnetic resonance (NMR) spectroscopy and gel permeation chromatography (GPC). Thermal stability and transitions were determined using thermogravimetric analysis (TGA), differential scanning calorimetry (DSC) and thermally stimulated depolarization current (TSDC). Furthermore, small angle X-ray scattering (SAXS) measurements and UV-Vis spectroscopy were performed to analyze temperature dependent transitions of the siloxanes and H-aggregates. A more detailed description can be found in the SI.

For thermally stimulated depolarization current (TSDC), dielectric relaxation spectroscopy (DRS), and pyroelectric measurements, samples cycle-DR1 and homo-DR1 were prepared with 100 µm spacers, thereby ensuring a constant thickness above the melting temperature. Block-DR1 was melt-pressed at temperatures of 130 °C for a duration of 6 h at 3 bars, using 200 µm spacers. The diameter of all measured samples was 1 cm.

To measure the pyroelectric (*p*) coefficient, a quasi-static periodic sinusoidal temperature variation was applied to a previously poled dielectric composite film (poling time = 10 min) using the Novocontrol Quatro cryosystem. A modulation frequency of 8.3 mHz and a temperature amplitude of 1 K were used for the measurements. The resulting current was measured using the Keysight B2985A electrometer. The samples were poled at 5 V µm^−1^ for 10 min at an initial temperature of 100 °C. The samples were then cooled to 0 °C while maintaining a constant voltage. After a 10 min poling period at 0 °C, the voltage was removed, and measurements were conducted for 1 h at 20, 35, and 60 °C.

Dielectric relaxation spectroscopy (DRS) was performed on a Novocontrol Alpha-A frequency analyzer at 1 V at frequencies between 10^−1^ and 10^6^ Hz. A Novocontrol Quatro cryosystem was used to control the sample temperature with a 2.5 K temperature step under a dry nitrogen atmosphere. For obtaining the derivative curves and fitting the dielectric data DCALC program developed by Wübbenhorst was used.^38,39^ Interdigitated electrodes were used from Novocontrol with an electrode diameter of 20 mm, an electrode basic structure size of 0.15 mm, a *C*_0_ of 5.01 pF, a pre-resistance of 0.1 Ohm, a resistance of the object carrier of 10^15^ Ohm, and a capacity of the object carrier of 15.2 pF.

The following reagents were used without further purification: 4-(dimethylamino)pyridine (DMAP) and *N*-(3-dimethylaminopropyl)-*N*′-ethylcarbodiimid hydrochlorid (EDC HCl) from Apollo Scientific; dry benzene, 2-bromoethanol from Fisher Scientific; hexamethyldisiloxane end-blocker (HM-EB), CaH_2_, tetramethylammonium hydroxide (TMAH) 25 wt% in H_2_O, 2,2-dimethoxy-2-phenylacetone (DMPA), mercaptopropionic acid, and disperse red 1 (DR1) from Sigma Aldrich; 1,3,5,7-tetramethyl-1,3,5,7-tetravinyl cyclotetrasiloxane (V_4_) and 1,3-bis(3-aminopropyl)tetramethyldisiloxan endblocker (NH_2_-EB) from ABCR; vinyl terminated polydimethylsiloxane, 200 cst *M*_w_ = 9400 g mol^−1^ from Geleste; and tetrahydrofuran, methanol, dichloromethane (DCM), and *n*-pentane from VWR.

### Synthesis of polymethylvinylsiloxane (PVS)

1,3,5,7-Tetramethyl-1,3,5,7-tetravinyl cyclotetrasiloxane (V_4_) and HM-EB were distilled over CaH_2_ (*T*_(V4,oil)_ 110 °C; *p* = 4.5 mbar), (*T*_(EB,oil)_ = 120 °C). TMAH (0.323 mL) was added to a dry three-necked flask equipped with a septum, a magnet stirring bar, and connected to the Schlenk line. TMAH was first dried under vacuum, followed by two azeotropic distillations with dry benzene. V_4_ (199.6 g, 200 mL, 0.579 mol, 1 eq.) and HM-EB (2.47 g, 3.23 mL, 0.015 mol, 0.026 eq.) were added, and the mixture was stirred for 1 h at room temperature. It was then heated to 80 °C and stirred for an additional 18 h. After the reaction was complete, the mixture was heated to 140 °C for 4 h to decompose TMAH. The product was purified by distilling unreacted reagents at 140 °C and 10^−2^ mbar overnight. ^1^H NMR (400 MHz, CDCl_3_, *δ*): 5.99 (m, 2H); 5.84 (m, 1H); 0.17 (m, 3H); 0.09 (s, HM-EB) (Fig. S1); ^13^C NMR (100 MHz, CDCl_3_, *δ*): 136.63; 133.06; 1.81 (HM-EB); −0.55 (Fig. S2). ^29^Si NMR (79 MHz, none, *δ*): end group: 7.03; cycles: −32.57; chains: −34.98 (Fig. S3), content of cycles of 6 wt%; GPC: *M*_n_ = 6.0 × 10^4^ g mol^−1^; *M*_w_ = 9.7 × 10^4^ g mol^−1^; *Đ* = 1.6 (Fig. S4).

### Synthesis of polymethylvinylsiloxane with aminopropyl end groups (H_2_N-PVS-NH_2_)

H_2_N-PVS-NH_2_ was synthesized analogously to PVS, replacing the HM-EB endblocker with 1,3-bis(3-aminopropyl) tetramethyldisiloxane. (More details see SI.) ^1^H NMR (400 MHz, CDCl_3_, *δ*): 5.96 (m, 2H); 5.81 (m, 1H); 2.69 (m, NH_2_-EB); 1.48 (m, NH_2_-EB); 0.54 (m, NH_2_-EB); 0.13 (m, 3H); 0.09 (s, NH_2_-EB) (Fig. S5); ^13^C NMR (100 MHz, CDCl_3_, *δ*): 136.63; 133.06; 1.81 (NH_2_-EB); −0.55 (Fig. S6). No GPC measurements were conducted because the amino groups on the polymer interact with the column.

### Synthesis of carboxylic acid end-functionalized PDMS (HOOC-PDMS-COOH)

Vinyl terminated PDMS (GPC: *M*_n_ = 2.5 × 10^4^ g mol^−1^; *M*_w_ = 3.3 × 10^4^ g mol^−1^; *Đ* = 1.4 (Fig. S7) (25.5 g, 2.71 mmol, 1 eq.), mercaptopropionic acid (1.15 g, 0.01 mol, 4 eq.), DMPA (0.067 g, 0.271 mmol, 0.1 eq.), and THF (250 mL) were added to a flask and degassed *via* the freeze–pump–thaw technique three times. Subsequently, the mixture was irradiated with a UV source for 15 min. The polymer was precipitated in H_2_O and methanol twice and subsequently dried. ^1^H NMR (400 MHz, CDCl_3_, *δ*): 2.80 (t, 4H); 2.70 (t, 2H); 2.62 (m, 8H); 2.52 (m, 2H); 0.89 (t, 4H); 0.08 (s, polymer chain) (Fig. S8); ^13^C NMR (100 MHz, CDCl_3_, *δ*): 1.19 (Fig. S9). GPC: *M*_n_ = 2.0 × 10^4^ g mol^−1^; *M*_w_ = 2.7 × 10^4^ g mol^−1^; *Đ* = 1.4 (Fig. S10).

### Synthesis of PVS-*block*-PDMS copolymer

H_2_N-PVS-NH_2_ (2.0 g, 0.28 mmol, 1 eq.), HOOC-PDMS-COOH (3.1 g, 0.28 mmol, 1 eq.), DMAP (0.137 g, 1.12 mmol, 4 eq.), and dry DCM (20 mL) were added into a dry Schlenk flask, and stirred for 5 min under Ar. Subsequently, EDC HCl (0.22 g, 1.2 mmol, 4 eq.) was added, and the mixture was stirred for two days at room temperature. The solution was precipitated in methanol and dried in a vacuum oven at 60 °C overnight. The ratio of methyvinylsiloxy to dimethylsiloxy was determined as 1 : 2.2 by using the integrals of the signals of the methyl and vinyl groups in the ^1^H NMR spectrum. ^1^H NMR (400 MHz, CDCl_3_, *δ*): 5.94 (m, 2H); 5.80 (m, 1H); 3.24 (q, linker); 2.81 (t, linker); 2.60 (t, linker); 2.43 (t, linker); 1.53 (m, linker); 0.91 (t, linker); 0.53 (t, linker); 0.14 (s, 3); 0.07 (s, 12.6) (Fig. S11); ^13^C NMR (100 MHz, CDCl_3_, δ): 136.57; 132.92; 0.86; -0.74 (Fig. S12).

### Synthesis of polysiloxane functionalized with carboxylic acid side groups (PS-COOH)

PVS (10.82 g, 0.126 mol repeating units, 1 eq.), mercaptopropionic acid (20 g, 0.188 mol, 1.5 eq. per RU), DMPA (0.168 g, 1.26 mmol, 0.01 eq. per RU), and THF (200 mL) were added to a flask and degassed *via* the freeze–pump–thaw technique three times. Subsequently, the mixture was irradiated with a UV source for 20 min. The polymer was precipitated in H_2_O and *n*-pentane. It should be noted that the polymer was not fully dried before the next step, as this would have increased viscosity and impeded the subsequent reaction. However, residual THF was permitted as it reduces viscosity, facilitating the next reaction step. 1H NMR (400 MHz, d-DMSO, *δ*): 12.22 (s, 1H); 2.70 (t, 2H); 2.58 (t, 2H); 2.52 (m, 2H); 1.29 and 0.88 (t, 2H); 0.17 (s, 3H) (Fig. S13); ^13^C NMR (100 MHz, d-DMSO, *δ*): 172.65; 33.87; 25.88; 25.17; 17.33; −0.81 (Fig. S14).

### Functionalization of PVS-*block*-PDMS with COOH groups (PS-COOH-*block*-PDMS)

PS-COOH-*block*-PDMS was synthesized analogous to PS-COOH (More details see SI) ^1^H NMR (400 MHz, d-DMSO, *δ*): only ranges can be given due to poor solubility of the material 2.92–2.4; 0.85; 0.01 (Fig. S15); ^13^C NMR could not be reported due to poor solubility.

### Synthesis of tetracyclosiloxane functionalized with carboxylic acid groups (Cycle-COOH)

Cycle-COOH was synthesized analogous to PS-COOH (More details see SI).

^1^H NMR (400 MHz, d-DMSO, *δ*): 12.22 (s, 1H); 2.67 (t, 2H); 2.55 (m, 2H); 2.48 (m, 2H); 1.24 and 0.85 (m, 2H); 0.13 (s, 3H) (Fig. S16); ^13^C NMR (100 MHz, d-DMSO, *δ*): 173.36; 34.50; 26.67; 25.85; 17.79; −0.17 (Fig. S17).

### Synthesis of DR1 functionalized silicone (homo-DR1)

PS-COOH (3.3 g, 1 eq. RU; 0.017 mol), dry THF (to achieve a solution of 0.1 g in 1 mL THF), DR1 (5.4 g, 1 eq., 0.017 mol), and DMAP (2.3 g, 1.1 eq.; 0.019 mol) were added to a dry Schlenk flask, and stirred for 30 min under Ar. EDC HCl (3.6 g, 1.1 eq.; 0.019 mol) was added, and the mixture was allowed to stir for one day at room temperature. The mixture was precipitated once in H_2_O and twice in methanol. Following purification, the polymer was dried in a vacuum oven at 60 °C overnight. ^1^H NMR (400 MHz, CDCl_3_, *δ*): 8.33–8.02 (2H); 7.94–7.64 (4H); 6.83–6.57 (2H); 4.34–4.14 (2H); 3.72–3.33 (4H); 2.90–2.49 (6H); 1.13 (3H) 0.87 (2H); 0.13 (3H) (Fig. S18), due to the low solubility, the signal-to-noise ratio was too high, to conduct ^13^C NMR. Content of cycles of 19 wt%. GPC: *M*_n_ = 3.3 × 10^4^ g mol^−1^; *M*_w_ = 5.2 × 10^4^ g mol^−1^; *Đ* = 1.6 (Fig. S19).

### Grafting DR1 to PS-COOH-*block*-PDMS (block-DR1)

Block-DR1 was synthesized analogous to homo-DR1 (more details see SI) ^1^H NMR (400 MHz, CDCl_3_, *δ*): 8.19 (2H); 7.79 (4H); 6.70 (2H); 4.23 (2H); 3.68 (2H); 3.49 (2H); 2.64 (2H); 2.5 (4H, covered by solvent) 1.12 (3H); 0.89 (2H); 0.07 (22.5H) (Fig. S20). No GPC and ^13^C NMR measurements were conducted due to the poor solubility of the sample.

### DR1 functionalized cyclosiloxane (cycle-DR1)

cycle-DR1 was synthesized analogous to homo-DR1 (more details see SI) ^1^H NMR (400 MHz, d-DMSO, *δ*): 8.33 (2H); 7.89 (2H); 7.80 (2H); 6.88–6.57 (2H); 4.34–4.14 (2H); 3.72–3.33 (4H); 2.90–2.49 (6H); 1.13 (3H) 0.78 (2H); 0.05 (3H) (Fig. S21). ^13^C NMR (100 MHz, d-DMSO, *δ*): 171.37; 156.12; 151.53; 146.83; 142.81; 125.9; 124.88; 122.43; 111.58; 61.56; 48.18; 44.91; 34.19; 25.95; 25.39; 17.18; 11.97; −0.70 (Fig. S22) GPC: *M*_n_ = 2.5 × 10^3^ g mol^−1^; *M*_w_ = 2.2 × 10^3^ g mol^−1^; *Đ* = 1.03 (Fig. S23).

The preparation and UV-Vis analysis of thin films is described in the SI.

## Results and discussion

### Synthesis and structural characterization

**Scheme 1:**
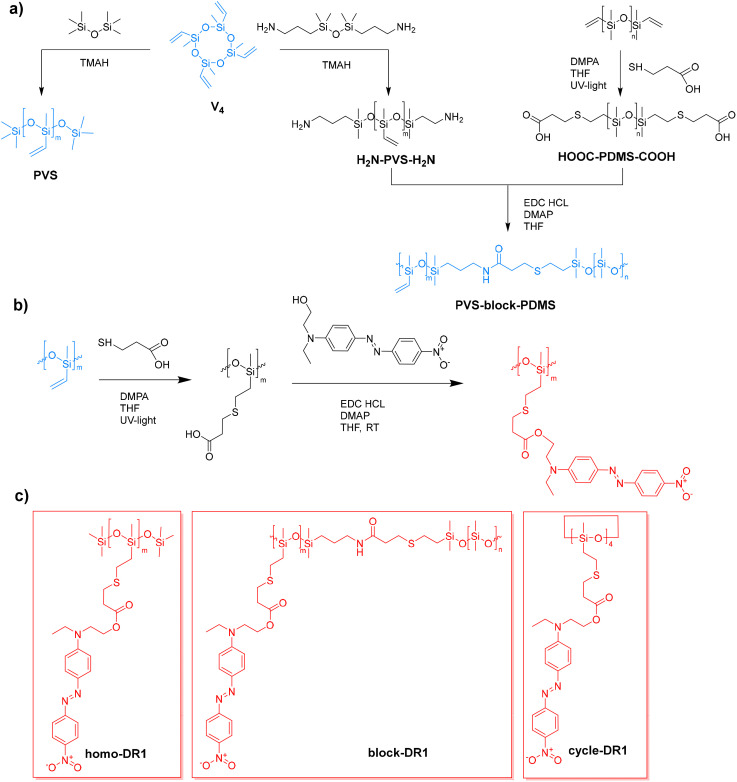
Synthesis route for the starting vinyl-containing homo and block polysiloxane (a). Synthetic strategy used for the functionalization of different siloxanes with DR1 group in two steps (b). First, the vinyl group is used in a thiol–ene click reaction with mercaptopropionic acid, followed by esterification with the DR1 moiety, which has a hydroxy group. Structures of the different DR1-functionalized siloxanes: homo-DR1, block-DR1, and cycle-DR1 (c).

Scheme 1 provides an overview of the synthetic steps used to obtain the desired silicones (homopolymer, block copolymers, and cyclic) modified with DR1 side groups. First, anionic ring-opening polymerization of V_4_ using TMAH as an initiator in the presence of either hexamethyldisiloxane or 1,3-bis(3-aminopropyl)tetramethyldisiloxane end blockers afforded PVS and H_2_N-PVS-NH_2_ (Scheme 1a). We aimed to obtain polysiloxanes with approximately 80 repeating units by adjusting the amount of end blocker used. As anticipated, ^1^H NMR analysis revealed a ratio of end groups to repeating units of 2 : 80, corresponding to a molecular mass of approximately 7100 g mol^−1^, as calculated by end-group analysis. The second block was a commercial PDMS that has vinyl end-groups (*M*_n_ = 9400 g mol^−1^ and *Đ* = 1.3 (Fig. S7)), which reacted with thiopropionic acid to give a PDMS with two carboxylic acid end groups (HOOC-PDMS-COOH). The average number of repeating units was calculated by ^1^H NMR and was found to be approximately 148, resulting in a *M*_n_ = 11 000 g mol^−1^, *Đ* = 1.4 (Fig. S5 and S10). The condensation reaction of equimolar H_2_N-PVS-NH_2_ and HOOC-PDMS-COOH yields the desired block copolymer, PVS-*block*-PDMS. The targeted ratio of methylvinylsiloxy to dimethylsiloxy was 80 : 148 (1 : 1.85), and the actual ratio obtained was 80 : 177 (1 : 2.21). Rheometry was used to verify the chain elongation. While the starting polymers exhibited relatively low shear stress (Fig. S24a), it increased by three orders of magnitude for PVS-*block*-PDMS.

In the next step, the vinyl groups of the three different starting materials (PVS, PVS-*block*-PDMS, and V_4_) were reacted using a thiol–ene click reaction with mercaptopropionic acid to introduce carboxylic acid side groups (Scheme 1b). They were subsequently used in a Steglich esterification with the DR1 moiety to give homo-DR1, block-DR1, and cycle-DR1, respectively.

GPC analysis of the homo-DR1 sample indicated a multimodal distribution (Fig. S19). The first peak exhibited the highest volume fraction of 77% with a *M*_n_ = 33 000 g mol^−1^, corresponding to around 65 RU. The low molar mass products consist of cyclic contaminants or linear oligomers.

Due to the high molar mass of PS-COOH-*block*-PDMS and its carboxylic acid functionality, the resolution of the ^1^H NMR spectrum was poor. Further evidence for the reaction was obtained by IR spectroscopy (Fig. S24b). PVS-*block*-PDMS exhibits a C–H bend from the vinyl group at 959 cm^−1^, a C=C stretch at 1601 cm^−1^, and a C–H stretch from the vinyl group at 3058 cm^−1^. Following the reaction, all bands associated with the vinyl group disappeared. In contrast, a broad band was observed above 3000 cm^−1^, which was attributed to the OH group. Additionally, a band at 1709 cm^−1^ was attributed to the C=O stretch, while a band at 1554 cm^−1^ was attributed to the OH bend. The changes observed in the IR spectra provide further evidence that the polymer has been fully functionalized with carboxylic acid groups.

In the final step, an esterification reaction was conducted to attach the DR1 dye. As the quality of the ^1^H NMR spectrum of the starting material was insufficient to permit the calculation of the ratio of RU_COOH_ to RU_SiOMe2_, it was assumed to remain constant after the chemical modification. After complete functionalization, the ratio of RU containing DR1 to RU_SiOMe2_ was approximately 1 : 3, indicating that polymer chains with a higher ratio of functional groups were lost during workup.

The complete functionalization of the cycles was confirmed by ^1^H NMR spectroscopy (Fig. S21 and S22). The cyclic compounds were analyzed by GPC (Fig. S23), and the elugram revealed three distinct fractions: a predominant fraction at low elution times, constituting 94% of the volume, followed by two smaller fractions of 2 and 4%, respectively. The first fraction exhibited a *M*_n_ of approximately 2100 g mol^−1^, which was attributed to the four-membered ring. The elution time and the *M*_n_ of this fraction were comparable to the second fraction observed in homo-DR1 (Fig. S19), thereby substantiating the hypothesis that the second largest fraction in homo-DR1 is indeed the cyclic species. The observed discrepancy in molecular mass can be attributed to the presence of both five- and four-membered rings in the homo-DR1 sample, leading to an elevated *M*_n_. The smaller fractions could not be adequately separated, however, they showed a *M*_n_ of 700 g mol^−1^, indicating the presence of tri and dimeric species.

### Thermal analysis and phase transitions

To assess the thermal stability of the materials, thermogravimetric analysis (TGA) was performed (Fig. S25). The materials exhibited thermal stability up to temperatures exceeding 200 °C, with a significant mass loss at 280 °C. A notable similarity in the mass-loss behavior of homo-DR1 and cycle-DR1 was observed, and both exhibit stronger degradation at lower temperatures than block-DR1. This can be attributed to the similar chemical composition of cycle-DR1 and homo-DR1, and to the earlier decomposition of the attached DR1 moiety, which is present at higher ratios in cycle-DR1 and homo-DR1 than in block-DR1.

**Fig. 1:**
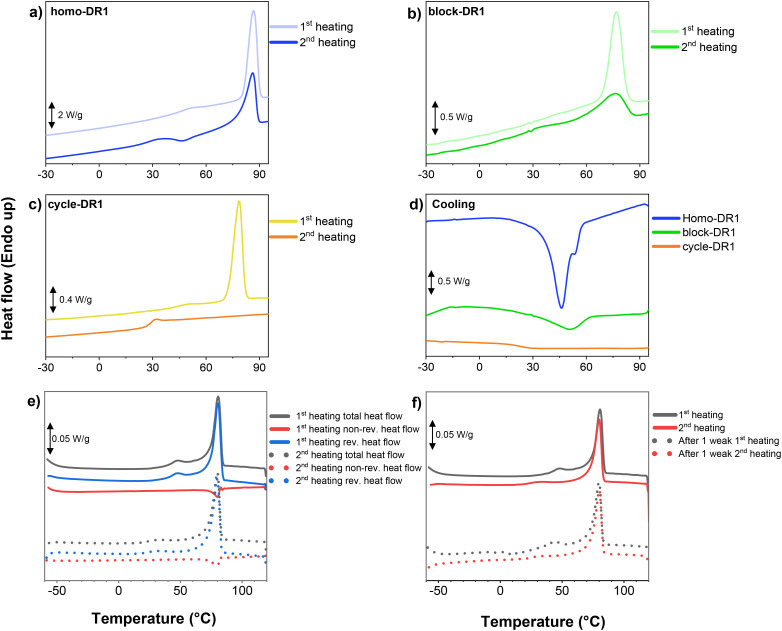
Differential scanning calorimetry of (a) homo-DR1 1st and 2nd heating; (b) block-DR1 1st and 2nd heating; (c) cycle-DR1 1st and 2nd heating; (d) cooling trace for all three samples; (e) modulated differential scanning calorimetry of homo-DR1; (f) differential scanning calorimetry of sample homo-DR1 before and after 1 week.

DSC was conducted on all three samples to investigate the phase transitions. For all samples, two transitions could be observed in the first heating cycle between −30 and 95 °C (Fig. 1a–c). A pronounced transition at higher temperatures of 87 °C (enthalpy of melting Δ*H*_m_ = 18.9 J g^−1^) for homo-DR1, 77 °C (8.7 J g^−1^) for block-DR1, and 78 °C (26.0 J g^−1^) for cycle-DR1. Additionally, a weaker thermal transition at lower temperatures around 50 °C in the homo-DR1 and block-DR1 samples, and 44 °C in the cycle-DR1 sample. The high temperature transition observed in all three samples can be attributed to the melting of primary DR1 crystalline regions (*T*_m,p_). The higher melting enthalpy of the cycle-DR1 sample suggests higher crystallinity than the other two samples, as expected, due to its greater tendency to crystallize, owing to the smaller number of repeating units and reduced flexibility. The weaker transition occurring at lower temperatures shifted down to a lower temperature during the second heating cycle to 37 °C for the homo- and block-DR1 and 31 °C for the cycle-DR1. Though during the initial heating cycle, this transition appears as an endothermic step associated with a typical glass transition, it appears as a broad peak during the second heating cycle in the homo- and block-DR1 samples. Hence, the first cooling curve of all three samples was plotted in Fig. 1d to obtain more information about this transition. While in the homo-DR1 we observe an exothermic recrystallization peak with a shoulder below 60 °C suggesting an additional crystallization step, the block-DR1 exhibited only a single weak exothermic peak around 50 °C. In addition, consistent with the absence of a melting peak in the 2nd heating curve of cycle-DR1, we do not observe a subsequent recrystallization peak in its corresponding cooling curve.

Modulated DSC (mDSC) measurements are useful in separating and identifying complex transitions. Fig. 1e shows the first two mDSC heating scans of a homo-DR1 sample. The endothermic peak in the non-reversible heat flow at 48 °C (first heating) and 33 °C (second heating) confirms the melting of secondary crystals, which have been observed in other semicrystalline materials.^40,41^ A similar behavior is observed in the block-DR1 and cycle-DR1 samples as well (Fig. S26 and S27). The lower heating rate used in mDSC leads to the observation of dual recrystallization peaks in the subsequent cooling cycle of the homo-DR1 sample (Fig. S28). However, we observe only a broad recrystallization peak for the other two samples (Fig. 1d). Secondary crystals are smaller than primary crystals and are usually formed during storage (aging) because they require longer crystallization times. As a result, their melting points (*T*_m,s_) and crystallization temperatures are strongly dependent on the sample's thermal history.^40,41^ This can be observed during the second heating cycle in Fig. 1e where the secondary crystallization peak becomes weaker and shifts to a lower *T*_m,s_. After allowing sufficient time (1 week), we observe that the *T*_m,s_ peak shifts back to the initial temperature recorded during the first heating scan (Fig. 1f). The extended time required for secondary crystal formation can also account for the absence of a secondary crystallization peak during cooling in block-DR1 and cycle-DR1 samples.

Concerning the primary melting peak, during the second heating scan, it becomes weaker and broader in the homo-DR1 and block-DR1 samples (Fig. 1a and b). This is reflected in the lower transition enthalpy of 15.1 J g^−1^ for homo-DR1 and 4.6 J g^−1^ for block-DR1 samples. In the cycle-DR1 sample, during the second heating, the *T*_m,p_ peak completely disappears from the recorded DSC curve and is only observed in the corresponding mDSC scan (Fig. S27) due to the lower heating rate. All these observations indicate that primary crystallization is also influenced by the samples' thermal history. In addition, we observe dual primary melting peaks (more clearly seen during the second heating) in Fig. S27, indicating different crystal sizes. During the second heating, an additional exothermic transition is observed at 60 °C, which can be associated with cold crystallization. Since this peak appears just before the two melting peaks, it can be inferred that the cold crystallization process leads to the formation of the bigger primary crystals. This phenomenon also explains the absence of a recrystallization peak during the cooling cycles (Fig. 1d and Fig. S27).

It should be noted that we do not observe a glass transition in either in the DSC or mDSC scans of the homo-DR1 and block-DR1 samples. On the other hand, in the cycle-DR1 sample, during the first cooling run, we observe a step between 5 and 30 °C, indicating a glass transition process (Fig. S27). A corresponding step is also observed in the reversible heat-flow signal within the same temperature range during the second heating run.

The SAXS profiles obtained during the heating–cooling–reheating (H–HC–HCH) protocol demonstrate the reversible formation of a lamellar nanostructure in homo-DR1 (Fig. S29a–c). During the initial heating (H), the reflections at *q* = 0.19, 0.38, and 0.57 Å^−1^ (1 : 2 : 3 ratio) confirm a lamellar periodicity with a long period *d* = 3.31 nm (2π/*q*_1_). Upon further heating, the progressive weakening and disappearance of the harmonic series at 90 °C indicates loss of long-range lamellar order; this temperature closely matches the DSC melting transition at 87 °C. During subsequent cooling (HC) and reheating (HCH), reappearance of the same reflection sequence shows that the lamellar morphology is thermally reversible, although possible small shifts in *q* may reflect minor hysteresis or thermal expansion. Structurally, the lamellar structure can be tentatively assigned to alternating sublayers in which the polysiloxane segments form the lamellar backbone, while the azo-aromatic DR1 residues create higher-electron-density layers, together defining the ∼3.3 nm repeat distance; this dimension is consistent with nanoscale segregation expected from the combined length of the siloxane spacer and pendant chromophore groups.

### Dielectric and polarization dynamics

**Fig. 2:**
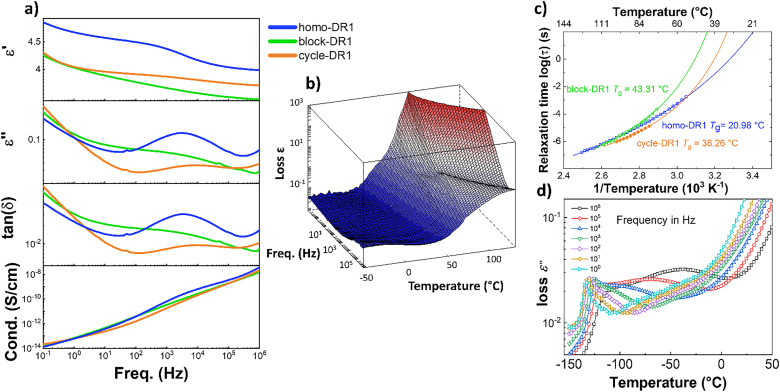
Impedance spectroscopy analysis of homo-DR1, block-DR1, and cycle-DR1: (a) dielectric permittivity *ε*′, dielectric losses *ε*″, tan(*δ*), and conductivity (*σ*) at room temperature; (b) 3D loss plots of homo-DR1 sample plotted as a function of frequency and temperature; (c) Arrhenius plot of the relaxation times obtained from Havriliak and Negami fits of the *T*_g_ relaxations observed in all three samples; (d) dielectric losses *ε*″ against temperature at selected frequencies for sample homo-DR1.

Dielectric relaxation spectroscopy (DRS) was used to analyze the three samples' dielectric properties. Fig. 2a shows the dielectric properties of the three measured samples as a function of frequency at room temperature (RT). The dielectric permittivity increases with decreasing frequency. The homo-DR1 sample showed a step increase in permittivity at a frequency around 3 kHz. This phenomenon is also evident in the other samples, albeit less intensely. The homo-DR1 displays the highest dielectric permittivity at 1 kHz, with a value of 4.4, followed by the cycle-DR1 with 3.9, and the block-DR1 with 3.7. As shown later in this section, the higher permittivity of the homo-DR1 sample at RT can be associated with its comparably lower *T*_g_, which is around RT, allowing the dipoles in the amorphous regions to be polarized. In comparison, the other two samples exhibit higher *T*_g_ values. Looking at the tan(*δ*) and *ε*″ plots, at high frequencies, we observe relaxation peaks in the homo-DR1 and cycle-DR1 samples at 3.1 kHz and 10 kHz, respectively, corresponding to the increase in permittivity. Within the same frequency range, the block-DR1 sample exhibits a broad transition, with a shoulder at approximately 5 kHz. All samples exhibit an increase in permittivity below 10 Hz, attributable to the onset of electrode polarization.^42^ The conductivity of the three samples at low frequency is rather low, below 10^−13^ S cm^−1^, confirming their excellent dielectric properties.

The dielectric properties of the samples were measured as a function of temperature to analyze the various transitions observed in detail. Fig. 2b shows the 3-dimensional loss plot of a homo-DR1 sample as a function of frequency and temperature. We observe an increase in losses above 10 °C, leading to relaxation peaks that shift to higher temperatures with increasing frequency. These loss peaks were fitted with the Havriliak–Negami function,^42^ and its corresponding relaxation map is plotted in Fig. 2c. The figure shows that this relaxation exhibits Vogel–Fulcher–Tammann (VFT) behavior, indicating a glass transition. The *T*_g_ can be calculated as the temperature at which the relaxation time is 100 s (log *τ* = 2 s).^42^ Accordingly, the homo-DR1 sample shows a *T*_g_ at 21 °C. From the 3D plot, we observe a sharp increase in losses around 50 and 70 °C. From the DSC/mDSC results, these increases in losses correspond to the melting of secondary and primary crystallites, respectively. Above 70 °C, once all crystals have melted, we observe increased losses at lower frequencies, due to electrode polarization. The low frequencies, combined with the elevated temperature above its *T*_g_, allow ions to migrate across the sample to the electrode-sample interface.

Fig. S30 and S31 show the 3D loss plots of the block-DR1 and cycle-DR1 samples, respectively. Both samples, similar to the homo-DR1 samples, exhibit frequency-independent loss peaks above 10 °C, which were fitted with the HN function We observe a VFT behavior with calculated *T*_g_s around 43 °C (block-DR1) and 36 °C (cycle-DR1) as shown in Fig. 2c. For the block-DR1, at temperatures below −100 °C, we observe an additional transition that also obeys the VFT law, yielding a *T*_g_ below −127 °C (Fig. S32), which is assigned to the *T*_g_ of the PDMS block.^43,44^ Just above the *T*_g_ relaxation, we observe yet another transition. This is better visualized in Fig. 2d where we observe the emergence of a shoulder at 100 Hz, which strengthens into a broad peak as frequency increases. This relaxation is also frequency dependent. However, fitting the peaks with the HN function results in a linear Arrhenius relaxation plot as shown in Fig. S32. This can be interpreted as interfacial polarization, commonly observed in multiphase materials. Ionic impurities present in the sample can be trapped at the interface.^42,45^ Above its *T*_g_, the PDMS chain segments are mobile, whereas the DR1 block remains frozen. This can cause the charges at these interfaces to relax, resulting in a Maxwell–Wagner interfacial (MWI) polarization.

Comparing the glass-transition temperatures of the three samples, the block-DR1 sample shows the highest *T*_g_, followed by the cycle-DR1 and the homo-DR1 sample. The higher *T*_g_ of cycle-DR1 compared to its corresponding homo-DR1 sample may be due to the higher crystallinity of the cycles, as indicated by the higher Δ*H*_m_ in DSC. With respect to the melting transitions, we observe a peak and a steep increase in losses around the same temperature at which we observe endothermic melting peaks in mDSC (Fig. S31). However, we do not observe such anomalies in the loss spectra of the block-DR1 sample, most likely due to the weak melting (Fig. S30).

**Fig. 3:**
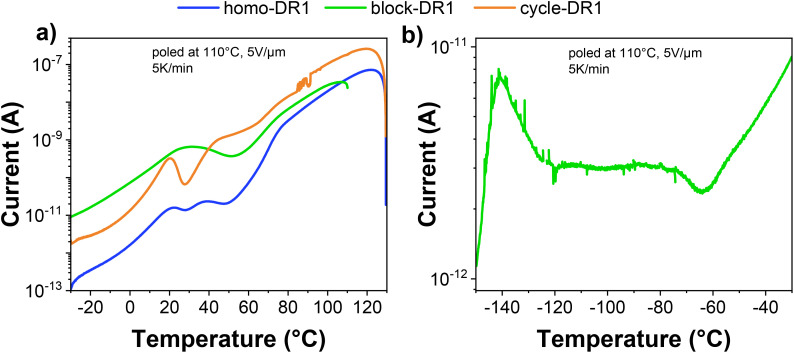
Thermally stimulated depolarization current measurement, the samples were poled for 10 min at 110 °C and 5 V µm^−1^ and measured at 5 K min^−1^: (a) sample homo-DR1, block-DR1, and cycle-DR1 in the region of −30 to 130 °C, and (b) sample block-DR1 in the region of −150 to −30 °C.

To complement the results from DSC and DRS, thermally stimulated depolarization currents (TSDC) measurements were performed on all samples (Fig. 3). TSDC is a sensitive thermal analysis of dielectrics where the sample is polarized at elevated temperatures above any transition temperature and subsequently cooled under a bias field to lock in the dipolar orientation. By monitoring the current discharge during a controlled heating ramp, specific molecular motions and structural relaxations can be observed. Thus, the sample's thermal history is erased prior to the start of the measurement, and the results obtained are comparable to those from the second heating cycle in DSC/mDSC. The heating rates employed in TSDC correspond to equivalent very low frequencies of 10^−3^ to 10^−4^ Hz in DRS measurements, and thus, the measurement is highly sensitive.

From Fig. 3, we can observe that sample homo-DR1 exhibited three discernible peaks at 22, 39, and 77 °C, respectively. Based on the results from DSC and DRS, we can assign these three peaks to *T*_g_, *T*_m,s_, and *T*_m,p_, respectively. In the case of cycle-DR1, the depolarization current exhibited three peaks over the same temperature range at 22, 40, and 70 °C. The peak at 40 °C is assigned to the glass transition, while those at 22 and 70 °C are assigned to *T*_m,s_ and *T*_m,p_, respectively. The block-DR1 sample exhibits a broad peak at 31 °C due to the superimposition of *T*_g_ and *T*_m,s_ transitions. At higher temperatures, we observe a sharp shoulder around 70 °C, corresponding to the melting of primary crystals. Fig. 3b shows the low-temperature TSDCs of the block-DR1 sample, which exhibits a peak at −140 °C due to the unfreezing of the PDMS blocks at the glass transition, followed by a broad transition between −120 and −70 °C, indicative of a MWI polarization. It has been demonstrated that certain azo dyes are capable of forming liquid crystals,^46,47^ a property that can manifest as a series of thermal transitions during the conversion process from one phase to another.^48^ To further investigate the potential occurrence of liquid crystals, the homo-DR1 sample was subjected to temperature-dependent polarized optical microscopy (POM) (Fig. S33). At temperatures above 80 °C, no structures can be observed in POM. At temperatures below 75 °C, crystal growth can be observed. However, upon further cooling to room temperature, no discernible alteration in the crystalline phase was evident. These findings imply the absence of liquid crystals within the polymer. However, temperatures below the second crystallization at 40 °C (Fig. 1d) resulted in the observation of low amounts of additional crystal formation. This phenomenon can be attributed to secondary crystals, as evidenced by thermal and dielectric measurements.

### H-Aggregates formation

Azo dyes are known for their ability to undergo *cis*-to-*trans* isomerization, with the *trans* state being more stable and having lower energy. The *cis* configuration can be accessed by supplying energy, such as UV light.^49,50^ DR1 also exists as dimers, known as H-aggregates, depending on the matrix and dye load.^51–53^ To examine the potential impact of these isomers and aggregates on existing systems, UV-Vis studies were conducted in both solution and thin-films. Solutions of our samples in THF exhibited a peak at 470 nm (Fig. S34). Exposure to UV for 5 min decreased the degree of absorption in the homo-DR1 and block-DR1 samples, accompanied by a slight blue shift, as evidenced by the appearance of an isosbestic point at 412 nm. In contrast, no discernible shift in the absorption spectrum was observed in cycle-DR1. The peak at 470 nm was assigned to the *trans* isomer, which has been observed as the dominant isomer in solution for all samples. However, exposure to UV light resulted in the formation of the *cis* isomer. This phenomenon has been documented in the literature. For example, Saibi *et al.* reported an isosbestic point for DR1 at 405 nm, as well as a decrease in absorption combined with a blue shift, which they attributed to the *trans* to *cis* isomerization.^54^

**Fig. 4:**
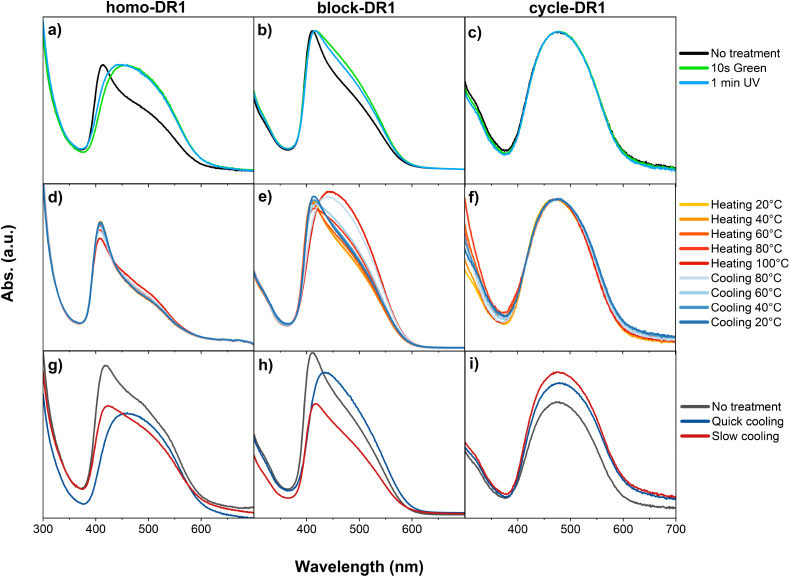
UV-Vis analysis of films made of homo-DR1 (left), block-DR1 (middle), and cycle-DR1 (right): (a)–(c) light-induced deaggregation; (d)–(f) heat-induced deaggregation; (g)–(i) influence of the cooling rate on aggregates.

All samples were spin-coated onto glass substrates to form thin films. The homo-DR1 and block-DR1 films exhibited an absorption maximum at 412 nm, followed by a shoulder at 490 nm. In contrast, cycle-DR1 exhibited only a maximum at 480 nm. A comparison of these values with those reported in the literature^52,54^ revealed that the peak at 412 nm can be assigned to H-aggregates of DR1. Meanwhile, the peak for cycle-DR1 and the shoulders at higher wavelengths for homo-DR1 and block-DR1 were assigned to the non-aggregated *trans* isomer. To test this hypothesis and exclude the possibility that the peak at lower wavelengths is due to the *cis*-isomer, all samples were irradiated with green light for 10 s. This resulted in a shift of the absorption maxima to 460 nm for homo-DR1 (Fig. 4a) and a notable increase in the shoulder at higher wavelengths for block-DR1 (Fig. 4b) compared to the untreated sample. Conversely, sample cycle-DR1 exhibited no alteration in the absorption spectrum (Fig. 4c). Subsequent irradiation with UV light resulted in a small blue shift for both polymer samples. In the case of block-DR1, the shoulder was less pronounced, while for homo-DR1, the peak shifted to 445 nm. However, for both polymer samples, the original spectra could not be reobtained. Sample cycle-DR1 showed no noticeable shift towards lower wavelength. Unlike in solution, both homo-DR1 and block-DR1 form H-aggregates in films. This observation suggests that, in solution or in solids, if sterically hindered, the non-aggregated *trans* configuration is strongly preferred. Conversely, in the solid state without sterically hinderance, as in homo-DR1 and block-DR1, aggregate formation is preferred. The input of energy in the form of green light results in the disintegration of the aggregates. Irradiation with UV light did not result in the initial spectrum. This suggests that the absorption of the initial spectrum is due to H-aggregates rather than a single *cis* or *trans* isomer. It is hypothesized that exposure to light provides sufficient energy to destroy the stacks. The differences observed between sample homo-DR1 and block-DR1 can be attributed to the varying thicknesses of the samples. The film containing block-DR1 was found to be significantly thicker, resulting in a better heat distribution and reducing destruction of the stacks, following the application of light. To determine whether the two distinct crystallization and melting peaks observed in DSC and TSDC (Fig. 1 and 3) are derived from the aggregated and non-aggregated DR1, temperature-dependent UV-Vis measurements were conducted on thin films (Fig. 4d and e). Samples homo-DR1 and block-DR1 showed red-shifted spectra upon heating to 100 °C. Conversely, a blue shift towards the initial spectrum was observed upon cooling to room temperature. In contrast, the cycle-DR1 showed no shift in its spectrum with changing temperature. These observations suggest that at elevated temperatures, the equilibrium shifts towards the non-aggregated DR1 form in both polymers. In contrast, at lower temperatures, the non-aggregated form is preferred only in sample cycle-DR1. The spectral change was predominantly observed at temperatures exceeding 40 °C for both polymers, with a gradual shift rather than an abrupt transition. Consequently, it can be deduced that temperatures above the *T*_g_, which is around 30 °C, are necessary for the transition from the aggregated to the non-aggregated DR1. A comparison of the previously discussed thermal transitions with the temperature-dependent spectra does not indicate a dependence of the H-aggregates on any of these transitions. While thermal transitions such as *T*_g_ and *T*_m_ occur in relatively well-defined, narrow temperature windows, the spectra exhibit no abrupt changes with temperature. Conversely, a gradual shift from aggregated to non-aggregated DR1 was observed with increasing temperature. As previously demonstrated by DSC (Fig. 1), the thermal history of the polymers is a significant factor influencing their crystallinity. The impact of different cooling rates on the aggregate formation was investigated. Without any treatment, both homo-DR1 and block-DR1 samples exhibit a peak of around 415 nm, while cycle-DR1 exhibits a peak at 480 nm. After melting and rapid cooling of all samples with liquid N_2_, red shifts in the absorption bands of homo-DR1 and block-DR1 were observed. However, after erasing the thermal history by heating above the melting temperature and subsequently cooling for over an hour, the spectra exhibited a blue shift and a shape similar to that of the initial sample (Fig. 4g and h). A higher proportion of H-aggregates was observed in the initial samples and the samples subjected to slow cooling. Conversely, the quenched samples exhibit a higher ratio of non-aggregated DR1. In contrast to homo-DR1 and block-DR1, the heating rate appears to have no discernible effect on the absorption spectra of sample cycle-DR1, which consistently displays a peak at 480 nm. As previously discussed (Fig. 4d and e), the equilibrium in homo-DR1 and block-DR1 shifts towards the non-aggregated DR1 in heated samples. Rapid cooling impedes DR1 aggregation, and below the *T*_g_, the non-aggregated DR1 moieties are frozen. The presence of either non-aggregated or aggregated DR1 explains the distinct spectra observed in the quenched and slowly cooled samples.

To verify the observed differences in the arrangement of the DR1 dye between the polymers and the cyclic species, the three samples were analyzed by FT-IR spectroscopy (Fig. S35a). Since the chemical composition of cycle-DR1 and homo-DR1 is the same, their IR spectra should be indistinguishable. In contrast, sample block-DR1 should exhibit partial chemical similarity to the other two samples, as it contains PDMS blocks and a linker. As anticipated, the IR spectra of the three samples demonstrated notable similarity. For sample cycle-DR1 the following bands were detected in the spectrum (Table 1): symmetric C–N stretch at 1128 cm^−1^; the asymmetrical stretch of the NO_2_ group at 1333 cm^−1^; the stretch of the N=N bond at 1383 cm^−1^; and the bands arising from the phenyl ring at 1585 and 1597 cm^−1^.^55,56^ These bands were also found in both polymers, however, they exhibited a slight change toward higher wavenumbers (Table 1 and Fig. S35b). One possible explanation for the observed phenomenon could be the aggregation of the DR1 dye. Aggregates have been identified in the sample block-DR1 and homo-DR1, both of which exhibit a shift in the bands toward higher wavenumbers. In contrast, sample cycle-DR1 shows no aggregation, and no shift to higher wavenumbers is observed.

**Table 1:** FTIR absorption spectroscopy of samples homo-DR1, block-DR1, and cycle-DR1. The wavenumbers of bonds that change with polymer architecture are compared

Group	homo-DR1 (cm^−1^)	block-DR1 (cm^−1^)	cycle-DR1 (cm^−1^)
Symmetrical C–N stretching	1130 (weak)	1133 (weak)	1128
Phenyl ring stretching	1587 (small)	1587 (small)	1585
	1599 (large)	1601 (large)	1597 (same height)
Azo stretching vibration	1387	1389	1383
Asym NO_2_ (symmetric)	1335	1338	1333
(asymmetric)	1512	1514	1512

As demonstrated by both the DSC (Fig. 1) and TSDC (Fig. 3), the functionalization of polysiloxanes with DR1 resulted in materials with a *T*_g_ and melting temperatures above or near room temperature. Consequently, the samples homo-DR1 and cycle-DR1 were too brittle to undergo tensile testing. However, the block-DR1 sample exhibited sufficient ductility for analysis (Fig. S36). The material exhibited a strain at break of 7.2% and its Young's modulus (*Y*_5%_) was 2.60 MPa. The stretchability is attributed to the PDMS block, which possesses a *T*_g_ well below room temperature (Fig. 3b). The comparatively high Young's modulus is due to increased crystallinity induced by the DR1-block.

### Pyroelectric performance

The quasi-static pyroelectric coefficient of all samples was measured for both the unpoled and poled samples. The unpoled samples of cycle-DR1 and block-DR1 exhibited a similar small pyroelectric response of about 0.12 µC m^−2^ K^−1^ at 60 °C, while the unpoled homo-DR1 exhibited about seven times higher pyroelectric response at the same temperature, indicating that homo-DR1 has spontaneous polarization. This spontaneous polarization, responsible for the pyroelectric response, could be due to the presence of H-aggregates or absorbed water in the sample. While aggregates can have a spontaneous dipole moment, water can also be absorbed by the material, leading to permanent polarization.^66^ As demonstrated by UV-Vis spectroscopy (Fig. 4), homo-DR1 and block-DR1 samples exhibit only a minor decrease in H-aggregate intensity when heated to 60 °C, indicating their contribution to the observed pyroelectricity. However, the cycle-DR1 contains no aggregates but still exhibits a pyroelectric response, suggesting that the adsorbed water may contribute to the measured effect. Of the three samples, homo-DR1 shows the strongest response of 0.83 µC m^−2^ K^−1^ (Fig. S37 and Table S1). Several samples were measured, and the responses were within the same range. The higher *p*-coefficient for the homo-DR1 sample may be attributable to the measurement temperature being much above its *T*_g_, leading to greater thermal expansion than in the other two samples. In addition, the block-DR1 contains a reduced amount of DR1 as compared to homo-DR1, while cycle-DR1 demonstrates a lower degree of H-aggregates. All these may lead to the lower *p*-coefficient observed in the block-DR1 and cycle-DR1cycle-DR1 samples.

Long-term measurements at 60 °C over 5 hours were recorded on the homo-DR1 sample. At the beginning of the measurement, a change in the current response was observed, with a slight decrease in the current magnitude over time. After 1 hour, the measured sample exhibited a pyroelectric coefficient of 1.47 µC m^−2^ K^−1^, and after 5 hours, it decreased to a rather stable value of 1.04 µC m^−2^ K^−1^ (Fig. S38).

To clarify the pyroelectric mechanisms in unpoled samples, the pyroelectric response of homo-DR1 and cycle-DR1 was measured at 60, 80, 100, and 120 °C (Fig. S39). Both samples exhibited increased pyroelectric responses with increasing temperature (Table S1), evidenced by rising current amplitudes. However, the response in cycle-DR1 was consistently lower than in homo-DR1 (Fig. S39a–d). This lower response, combined with the absence of H-aggregates in cycle-DR1 (UV-Vis, Fig. 4), suggests the response stems from an alternative factor, likely absorbed water. While water may also contribute to the homo-DR1 pyroelectric response, the significantly higher current generated is primarily attributed to spontaneous polarization due to aggregation. Despite cycle-DR1 and homo-DR1 having similar DR1 content and hygroscopic capacity, homo-DR1's architecture more readily promotes aggregate formation.^49^

Although we were unable to conduct pyroelectric measurements across different relative humidities due to equipment limitations, we nevertheless investigated the influence of water on the pyroelectric effect by preparing a homo-DR1 sample on interdigitated electrodes. This setup enabled us to measure films 10 µm thick without a top metal electrode, facilitating efficient water removal *via* heating, while keeping the sample under inert nitrogen gas flow at all times. The pyroelectric response of homo-DR1 was first measured at 60 °C, followed by 3 hours measurement at 120 °C, to ensure water was removed, and again at 60 °C (Fig. S40) to compare it with the undried measurement conducted earlier. In the initial measurement, prior to drying, changes in the pyroelectric response are observed; after approximately 1000 s, the response stabilizes. Subsequent drying and cooling and re-measurement at 60 °C revealed comparable current amplitudes before (4.65 × 10^−11^ A) and after thermal treatment (5.11 × 10^−11^ A) (Fig. S40a). These results suggest that thermal annealing stabilizes the pyroelectric response.

**Fig. 5:**
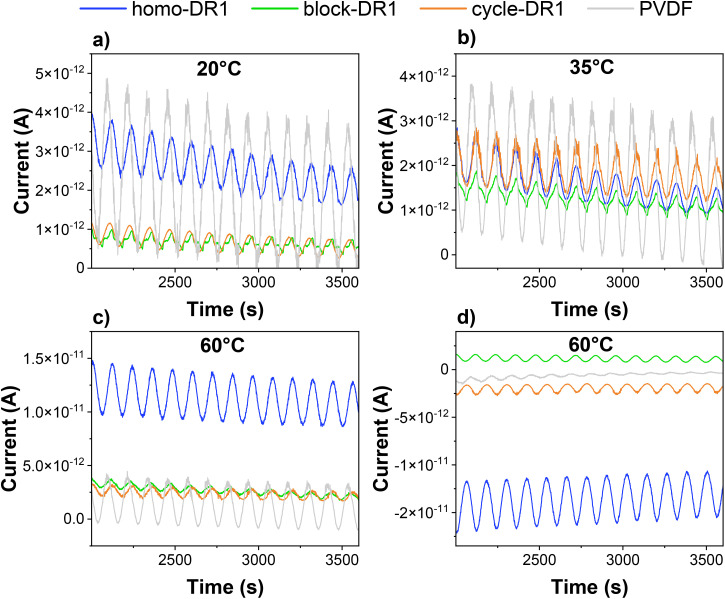
Pyroelectric measurement at 20 °C (a), 35 °C (b), and 60 °C (c) of sample homo-DR1, block-DR1, cycle-DR1, and PVDF poled at 100 °C with an electric field of −5 V μm^−1^. Pyroelectric response of unpoled samples at 60 °C (d).

The persistent pyroelectric current of the homo-DR1 sample at 120 °C, above its *T*_m_, points to the contribution from aggregates, as confirmed from UV-vis results (Fig. 4). It is to be mentioned that there could be an additional contribution from residual water, if present, that could be strongly bound in the aggregates. However, as stated previously, its contribution is expected to be much lower than that from aggregates. As shown in Fig. S40b, the current amplitude remains constant over three hours at 120 °C, demonstrating the sample's exceptional long-term thermal stability. To evaluate the performance of our unpoled material, the pyroelectric response of PVDF was measured under identical conditions. As shown in Fig. 5d, at 60 °C, the unpoled PVDF film exhibits a pyroelectric response, though it is significantly weaker than that of the homo-DR1. Moreover, the signal's poor stability suggests the presence of moisture, which evaporates during testing, weakening the observed effect. The pyroelectric response can be increased by poling in an electric field. The polar DR1 crystals, present in all samples, can be polarized by applying an electric field above their *T*_m_ and cooling the samples down to room temperature (below their *T*_g_) while the field is on. The resulting samples should exhibit both primary and secondary pyroelectricity at RT arising from the frozen dipoles in the crystalline and amorphous regions, respectively.^16^ Hence, the samples were poled at 100 °C and cooled subsequently to 20 °C under a DC field of −5 V µm^−1^. The polarized samples were subjected to quasi-static pyroelectric measurements at 20, 35, and 60 °C, thereby enabling investigation of the effects of the different thermal transitions on the materials' pyroelectric coefficient (*p*). The resulting (*p*) values and pyroelectric current are reported in Table 2 and Fig. 5, respectively. While the unpoled samples show no pyroelectric response at 20 and 35 °C, the poled samples exhibit a pyroelectric response. At 60 °C, above the sample's *T*_g_, the strongest response was observed for homo-DR1, but surprisingly, the pyroelectric response was not higher than that of the unpoled samples, which may be due to the rather low poling electric field, insufficient to orient the crystalline dipoles. However, the dipoles maintain their orientation up to 60 °C and are not affected by phase transitions, such as the glass transition and the melting of secondary crystals. For both polymeric species (homo-DR1 and block-DR1), the pyroelectric effect increases with increasing temperature from 35 to 60 °C, while the pyroelectric response for cycles remains constant at the measured temperatures and is not affected by poling. This finding suggests that thermal expansion, which increases as it passes the *T*_g_ and the melting of the secondary crystals, is responsible for the increased response *via* the secondary pyroelectric effect.

**Table 2:** Comparison of the pyroelectric coefficient of the three different samples, depending on the measurement temperature and the poling conditions, measured in the same sample

Sample	*p*-Coefficient (µC m^−2^ K^−1^) (unpoled)			*p*-Coefficient (µC m^−2^ K^−1^) (poled: 5 V µm^−1^)		
	20 °C	35 °C	60 °C	20 °C	35 °C	60 °C
homo-DR1	—	—	0.83 ± 0.43	0.21	0.12	0.66
block-DR1	—	—	0.12	0.03	0.06	0.11
cycle-DR1	—	—	0.11	0.12	0.12	0.11
PVDF	—	—	—	0.94	0.86	1.15

Compared to commercial PVDF under identical poling conditions, the synthesized materials exhibit lower pyroelectric response at low temperatures. However, at 60 °C, the difference between homo-DR1 sample and PVDF is reduced. Table 3 lists the pyroelectric coefficient of various fluorinated and non-fluorinated polymers found in literature. As shown in the table, the measured pyroelectric coefficient of PVDF strongly depends on the sample's processing conditions, poling field, and measurement temperature. Poling in higher fields increases the pyroelectric response because more dipoles are polarized. Our attempt to pole the homo-DR1 at higher electric fields failed due to dielectric breakdown. Increasing the dielectric breakdown strength of these polymers could further enhance the pyroelectric response. Though homo-DR1 shows a lower pyroelectric response than fluorinated polymers, it shows a comparable response with respect to other non-fluorinated polymers, which were all poled at much higher electric fields. The advantage of homo-DR1 is that it exhibits spontaneous pyroelectricity without the need for a poling step. Table 4 compares the spontaneous pyroelectric response of the homo-DR1 sample with that of two other recently reported polymers. While the PVDF sample exhibits a higher *p*-value, it is be noted that the sample was subjected to a specialized processing step that stretches the semi-cured polymer film and orients its dipole during fabrication.^64^ On the other hand, the homo-DR1 sample does not require any additional processing as the stacks are intrinsically formed in the homopolymer. With respect to the other reported zwitterionic polyelectrolyte brushes, homo-DR1 shows a higher spontaneous pyroelectric current, though at a higher temperature. Hence, the homo-DR1 samples are better suited for micro-energy harvesting at elevated temperatures, such as those from waste heat sources. To improve the properties of the novel DR1-functionalized polymers reported in this work, future research should focus on enhancing their dielectric breakdown strength. This may be achieved through filler incorporation, increased cross-linking density, or the fabrication of thin films.

**Table 3:** Comparison of pyroelectric coefficient of various fluorinated and non-fluorinated polymers along with their poling field and measurement temperature

Sample	*p*-Coefficient (µC m^−2^ K^−1^)	Poling field & measurement temperature
homo-DR1	0.66	5 V µm^−1^ at 60 °C
Cast PVDF film	1.15	5 V µm^−1^ at 60 °C
Spin-coated PVDF^19^	∼30	14 V µm^−1^ at 25 °C
Spin-coated PVDF^57^	92	80 V µm^−1^ at 35 °C
Polyvinylidene fluoride-trifluoroethylene^58^	30	200 V µm^−1^ at 25 °C
Polyvinylfluoride^59^	12–16	200 V µm^−1^
Polyvinylchloride^60^	1.0	170 V µm^−1^ at 25 °C
Polynorbornene-DR1^16^	1.23	25 V µm^−1^ at 25 °C
Azobenzene alkoxy-substituted polyvinyl alcohol^61^	0.2	25 V µm^−1^ at 25 °C
Polyacrylonitrile-*co*-vinylacetate^62^	1.94	30 V µm^−1^ at 70 °C
Nitroaniline-modified thermoplastic polyurethane^63^	1.30	30 V µm^−1^ at 25 °C

**Table 4:** Comparison of the spontaneous pyroelectric coefficient and current of reported polymers

Sample	*p*-Coefficient (µC m^−2^ K^−1^)	Pyroelectric current [pA]
homo-DR1	0.83 at 60 °C	4.5 at 60 °C
PVDF^64^	12.4 at 25 °C	—
Zwitterionic polyelectrolyte brushes^65^	—	0.85 & 2.37 at 25 °C

## Conclusions

In this study, we investigated the structure–property relationships of three siloxanes functionalized with DR1 moieties: a cyclosiloxane, a homopolymer, and a block copolymer. DSC analysis revealed two melting transitions at or above room temperature for all materials, while TSDC and DRS measurements identified characteristic glass transition temperatures. As expected, the block copolymer exhibited two distinct *T*_g_ values, one for each block. At room temperature, the low dielectric permittivity across all samples indicates “frozen” dipole. However, above the *T*_g_, the dipoles' mobility increases, enabling the induction of permanent polarization *via* poling at 100 °C. Among the poled samples, homo-DR1 exhibited the superior pyroelectric response, reaching 0.66 µC m^−2^ K^−1^ at 60 °C, attributed to its higher crystallinity and lower *T*_g_. Intriguingly, all three unpoled samples exhibited spontaneous polarization and thus pyroelectricity at 60 °C. UV-Vis spectroscopy suggests this behavior stems primarily from molecular aggregates, with possible additional secondary contribution from absorbed water. The comparable pyroelectric response of poled and unpoled samples at 60 °C suggests that the pyroelectric response is dominated by secondary pyroelectricity (thermal expansion/contraction of the polymer). These findings underscore that polymer architecture is a decisive factor in engineering high-performance dielectric and pyroelectric materials using DR1 chromophores.

## Author contributions

M. B. conducted the synthesis and structural characterization of all compounds and most measurements. F. T. conducted the DCS measurements. The analysis of the impedance spectroscopy data was conducted by T. R. V. Small-angle X-ray scattering measurements were conducted and interpreted by L. B. and R. M. M. B., T. R. V., and D. M. O. wrote the first version of the manuscript with input from all authors. D. M. O. initiated the activity, received funding, and coordinated and supervised this research. All authors contributed to discussions and approved the final version of this manuscript.

## Conflicts of interest

There are no conflicts to declare.

## Supplementary Material

MH-013-D6MH00410E-s001

## Data Availability

The data supporting the findings of this study are available within the article and its supplementary information (SI). Supplementary information: 1H, 13C, 29Si NMR spectra, FTIR spectra, GPC elugrams, UV-Vis spectra, dielectric spectroscopy data, DCS curves, TGA, and pyroelectric investigations. See DOI: https://doi.org/10.1039/d6mh00410e. The raw data generated and analyzed during the current study are available from the corresponding author upon reasonable request. All raw data were uploaded to Zenodo: 10.5281/zenodo.18864358 and will be made available on request.
