# Supplementary material for: Synthesis and pyroelectric response of disperse red 1 functionalized silicones: cyclic monomer, homopolymer, and block copolymer derivatives
Source: Mater Horiz. 2026 May 4;13(13):6553–68. doi: 10.1039/d6mh00410e (PMC13158845; doi:10.1039/d6mh00410e)
Supplement: MH-013-D6MH00410E-s001 [file MH-013-D6MH00410E-s001.pdf]

Supplementary Information (SI) for Materials Horizons.
This journal is © The Royal Society of Chemistry 2026

Synthesis and Pyroelectric Response of Disperse Red 1 Functionalized Silicones: Cyclic Monomer, Homopolymer, and Block Copolymer Derivatives

Malte Sebastian Beccard,[a,b] Thulasinath Raman Venkatesan,[a*] Francesco Taddei,[c] Laura Baraldi,[d] Raffaele Mezzenga,[d] Frank A. Nüesch,[a,e] and Dorina M. Opris[a,b*]

Experimental

^{1}H, ^{13}C, and ^{29}Si nuclear magnetic resonance (NMR) spectra were recorded on a Bruker Avance 400 NMR spectrometer at 298 K using a 5 mm broadband probe at 400.18 and 100.63 Hz. Chemical shifts are given relative to the solvents ($CHCl_3$: δ = 7.26 ppm and 77.16 ppm; DMSO: δ = 2.50 ppm and 39.52 ppm). A 0.1 M chromium(III) acetylacetonate solution in $CDCl_3$ is utilized as a paramagnetic relaxation agent in ^{29}Si NMR measurements. The resulting chemical shifts are given relative to TMS (δ = 0 ppm).

Thermogravimetric analysis (TGA) was performed on a Perkin Elmer TGA8000, heating the samples from 30 to 600 °C at a rate of 20 °C min^{-1} under air.

Differential scanning calorimetry (DSC) was performed on a Perkin Elmer DSC8000 over the temperature range of -80 to 100 °C with a heating/cooling rate of 20 °C min^{-1} under N_2. Modulated DSC measurements were carried out using a Netzsch DSC 204F1 Phoenix setup. The samples were heated and cooled at a linear rate of 2.5 K min^{-1}, with an additional modulation amplitude of ±0.21 K applied every 40 s only during heating.

For thermally stimulated depolarization current (TSDC), dielectric relaxation spectroscopy (DRS), and pyroelectric measurements, samples cycle-DR1 and homo-DR1 were prepared with 100 µm spacers, thereby ensuring a constant thickness above the melting temperature. Block-DR1 was melt-pressed at temperatures of 130 °C for a duration of 6 h at 3 bars, using 200 µm spacers. The diameter of all measured samples was 1 cm.

To measure the pyroelectric (p) coefficient, a quasi-static periodic sinusoidal temperature variation was applied to a previously poled dielectric composite film (poling time = 10 min) using the Novocontrol Quatro cryosystem. A modulation frequency of 8.3 mHz and a temperature amplitude of 1 K were used for the measurements. The resulting current was measured using the Keysight B2985A electrometer. The samples were poled at 5 V µm^{-1} for 10 min at an initial temperature of 100 °C. The samples were then cooled to 0 °C while maintaining a constant voltage. After a 10 min poling period at 0 °C, the voltage was removed, and measurements were conducted for 1 h at 20, 35, and 60 °C.

Small Angle X-ray Scattering (SAXS) measurements were performed on a Bruker AXS Micro, with a microfocused X-ray source, operating at a voltage and filament current of 50 kV and 1000 µA, respectively. The Cu Kα radiation (λCu Kα = 1.5418 Å) was collimated by a 2D Kratky collimator, and the data were collected by a 2D Pilatus 100 K detector. The scattering vector Q = (4π/λ) sin θ, with 2θ being the scattering angle, was calibrated using silver behenate. Data were collected and azimuthally averaged using the Saxsgui software to yield 1D intensity vs. scattering vector Q, with a Q range from 0.001 to 0.5 Å–1. For all measurements, the powder sample was placed inside a quartz capillary cell with a sample volume of 100 µL and a thickness of ~1,5 mm. Samples were equilibrated for 60 min prior to each measurement, and the scattering intensity was collected for 15 min.

Tensile tests of dog-bone-shaped samples with a width of 2 mm and a length of 18 mm were performed on a Zwick Z010 machine. The applied preload force was 0.005 N, and the sample was stretched at 50 mm min^{-1}. To determine the average, at least five samples were measured, and Young's modulus was determined by linearly fitting the data points from 0 to 5% strain.

UV-Vis measurements were conducted with a Varian Cary 50 UV-Vis spectrophotometer over 300-800 nm. As a UV-source 6 LEDs from Distrelec with a wavelength of 365 nm and a radiant power of 1 W per LED were mounted on a printed circuit board (PCB) plate on a surface of 2 cm^{2}. For the green light, 6 LEDs from Distrelec, with a wavelength of 505 nm and a radiant power of 1.1 W, were mounted on a PCB plate on a surface of 2 cm^{2}.

Gel permeation chromatography (GPC) measurements were performed using an Agilent 1100 Series HPLC (columns: serial coupled PSS SDV 5 m, 100 Å and PSS SDV 5 m, 1000 Å, detector: DAD, 235 nm and 360 nm; refractive index). THF (1 mL min^{-1}, 35 °C) was used as the mobile phase. Polystyrene (PS) standards were used to estimate the molar masses.

Thermally stimulated depolarization current (TSDC) measurements were conducted using a Keysight electrometer with a built-in DC voltage source. The films were poled at 110 °C using a 5 V μm^{-1} electric field for 10 min. Thereafter, the temperature decreased to -30 °C (-130 °C for **block-DR1**) while the voltage was maintained constant. The films were then heated at a rate of 5 K min^{-1} using a Novocontrol Quatro cryosystem under a nitrogen atmosphere.

Dielectric relaxation spectroscopy (DRS) was performed on a Novocontrol Alpha-A frequency analyzer at 1 V at frequencies between 10^{-1} and 10^{6} Hz. A Novocontrol Quatro cryosystem was used to control the sample temperature with a 2.5 K temperature step under a dry nitrogen atmosphere. For obtaining the derivative curves and fitting the dielectric data DCALC program developed by Wübbenhorst was used.[38,39] Interdigitated electrodes were used from Novocontrol with an electrode diameter of 20 mm, an electrode basic structure size of 0.15 mm, a C_0 of 5.01 pF, a pre-resistance of 0.1 Ohm, a resistance of the object carrier of 10^{15} Ohm, and a capacity of the object carrier of 15.2 pF.

Preparation of thin films

DR1 functionalized silicone was placed between two Teflon-coated metal plates separated by 100 µm thick spacers. The sample was pressed at 3 bars for 3 min at 110 °C. The **block-DR1** sample was pressed for 4 hours at 110 °C and 3 bars, then cooled while remaining in the press.

UV-Vis spectroscopy

A small amount of a solution of **homo-DR1** or **cycle-DR1** in DCM or **block-DR1** in NMP was spin-coated onto a glass substrate. After complete solvent evaporation, the glass substrate was placed on a heating stage suitable for UV-Vis spectroscopy.

Synthesis

Synthesis of polymethylvinylsiloxane with aminopropyl end groups (H_2N-PVS-NH_2)

V_4 was distilled over CaH_2 ($T_{(V4,oil)}$ 110 °C; p = 4.5 mbar). A solution of TMAH (0.1 mL) was introduced into a dry three-necked flask equipped with a septum, a magnet stirring bar, and a connection to the Schlenk line. The TMAH was first dried under vacuum, followed by two azeotropic distillations with dry benzene. V_4 (39.9 g, 40 mL, 0.116 mol, 1 eq.) and 1,3-bis(3-aminopropyl)tetramethyldisiloxan end blocker (1.44 g, 1.6 mL, 5.79 mol, 0.05 eq.) were added, and the mixture was stirred 1 h at room temperature. Subsequently, the mixture was heated to 80 °C and stirred for 18 h. Upon completion of the reaction, the mixture was heated to 140 °C for 4 h to decompose the TMAH. The product was purified by distilling the unreacted reagents at 140 °C and 10^{-2} mbar overnight. ^{1}H NMR (400 MHz, $CDCl_3$, δ): 5.96 (m, 2H); 5.81 (m, 1H); 2.69 (m, NH_2-EB); 1.48 (m, NH_2-EB); 0.54 (m, NH_2-EB); 0.13 (m, 3H); 0.09 (s, NH_2-EB) (**Fig. S5**); ^{13}C NMR (100 MHz, $CDCl_3$, δ): 136.63; 133.06; 1.81 (NH_2-EB); -0.55 (**Fig. S6**). No GPC measurements were conducted because the amino groups on the polymer interact with the column.

Functionalization of PVS-*block*-PDMS with COOH groups (PS-COOH-*block*-PDMS)

PVS-*block*-PDMS (3.21 g, 0.0386 mol repeating units, 0.0121 mol vinyl repeating units, 1eq.), mercaptopropionic acid (1.92 g, 0.0181 mol, 1.5 eq. per vinyl-RU), DMPA (0.154 g, 0.6 mmol, 0.05 eq. per vinyl-RU), and THF (32 mL) were added to a flask. The mixture was degassed by applying the freeze-pump-thaw technique three times. The reaction was then conducted under a UV irradiation source for 15 min. The polymer was precipitated from H_2O and dissolved in THF, a process that appeared challenging due to the polymer's poor solubility. It was assumed that the ratio of carboxylic acid functionalized repeating units to dimethylsiloxy repeating units was 1:2.2. ^{1}H NMR (400 MHz, *d*-DMSO, δ): only ranges can be given due to poor solubility of the material 2.92 – 2.4; 0.85; 0.01 (**Fig. S15**); ^{13}C NMR could not be reported due to poor solubility.

Synthesis of tetracyclosiloxane functionalized with carboxylic acid groups (Cycle-COOH)

$\mathbf{V_4}$ (15.5 g, 0.045 mol, 1 eq.), mercaptoproprionic acid (21.48 g, 0.203 mol, 4.5 eq.), DMPA (0.58 g, 2.2 mmol, 0.05 eq.), and THF (200 mL) were added to a flask. The flask was degassed three times via the freeze-pump-thaw technique. Subsequently, the mixture was exposed to UV light for 15 min. The mixture was subsequently concentrated using a rotary evaporator and washed with water three times. After complete purification, the cycles were dried overnight in the vacuum oven at 60 °C. ^{1}H NMR (400 MHz, *d*-DMSO, δ): 12.22 (s, 1H); 2.67 (t, 2H); 2.55 (m, 2H); 2.48 (m, 2H); 1.24 and 0.85 (m, 2H); 0.13 (s, 3H) (**Fig. S16**); ^{13}C NMR (100 MHz, *d*-DMSO, δ): 173.36; 34.50; 26.67; 25.85; 17.79; -0.17 (**Fig. S17**).

Grafting DR1 to PS-COOH-*block*-PDMS (block-DR1):

PS-COOH-*block*-PDMS (3.2 g, 0.029 mol RU, 0.009 mol carboxylic acid functionalized RU), dry THF (32 mL to achieve a solution of 0.1 g in 1 mL THF), DR1 (2.84 g, 9 mmol, 1 eq.), and 4-DMAP (1.1 g, 9 mmol, 1 eq.) were given into a dry Schlenk flask, and stirred for 30 min under Ar. EDC HCl (2.84 g, 9 mmol, 1 eq.) was added, and the mixture was allowed to stir for one day at room temperature. ^{1}H NMR spectroscopy was used to verify the complete reaction of the DR1 by monitoring the disappearance of the peak from the OH-group at 4.8 ppm in *d*-DMSO. If after 1 day, there was still a peak from the OH-group visible, more EDC HCl and DMAP were added. The mixture was precipitated once in H_2O and twice in methanol. After purification, the polymer was dried in the vacuum oven at 60 °C overnight.

^{1}H NMR (400 MHz, $CDCl_3$, *δ*): 8.19 (2H); 7.79 (4H); 6.70 (2H); 4.23 (2H); 3.68 (2H); 3.49 (2H); 2.64 (2H); 2.5 (4H, covered by solvent) 1.12 (3H); 0.89 (2H); 0.07 (22.5H) (**Fig. S20**). No GPC and ^{13}C NMR measurements were conducted due to the poor solubility of the sample.

DR1 functionalized cyclosiloxane (cycle-DR1)

Cycle-COOH (1.25 g, 1.6 mmol, 1 eq.), dry THF (13 mL to achieve a solution of 0.1 g in 1 mL THF), and DMPA (0.87 g, 6.5 mmol, 4.4 eq.) were added to a dry Schlenk flask, and stirred for 30 min under Ar. EDC HCl (1.37 g, 7.1 mmol, 4.4 eq.) was added, and the mixture was stirred overnight at RT. The mixture was concentrated and precipitated three times in methanol. ^{1}H NMR (400 MHz, *d*-DMSO, *δ*): 8.33 (2H); 7.89 (2H); 7.80 (2H); 6.88 – 6.57 (2H); 4.34 – 4.14 (2H); 3.72 – 3.33 (4H); 2.90 – 2.49 (6H); 1.13 (3H) 0.78 (2H); 0.05 (3H) (**Fig. S21**). ^{13}C NMR (100 MHz, d-DMSO, δ): 171.37; 156.12; 151.53; 146.83; 142.81; 125.9; 124.88; 122.43; 111.58; 61.56; 48.18; 44.91; 34.19; 25.95; 25.39; 17.18; 11.97; -0.70 (**Fig. S22**) GPC: M_n = 2.5×10^3 g mol^{-1}; M_w = 2.2×10^3 g mol^{-1}; $Đ$ = 1.03 (**Fig. S23**).

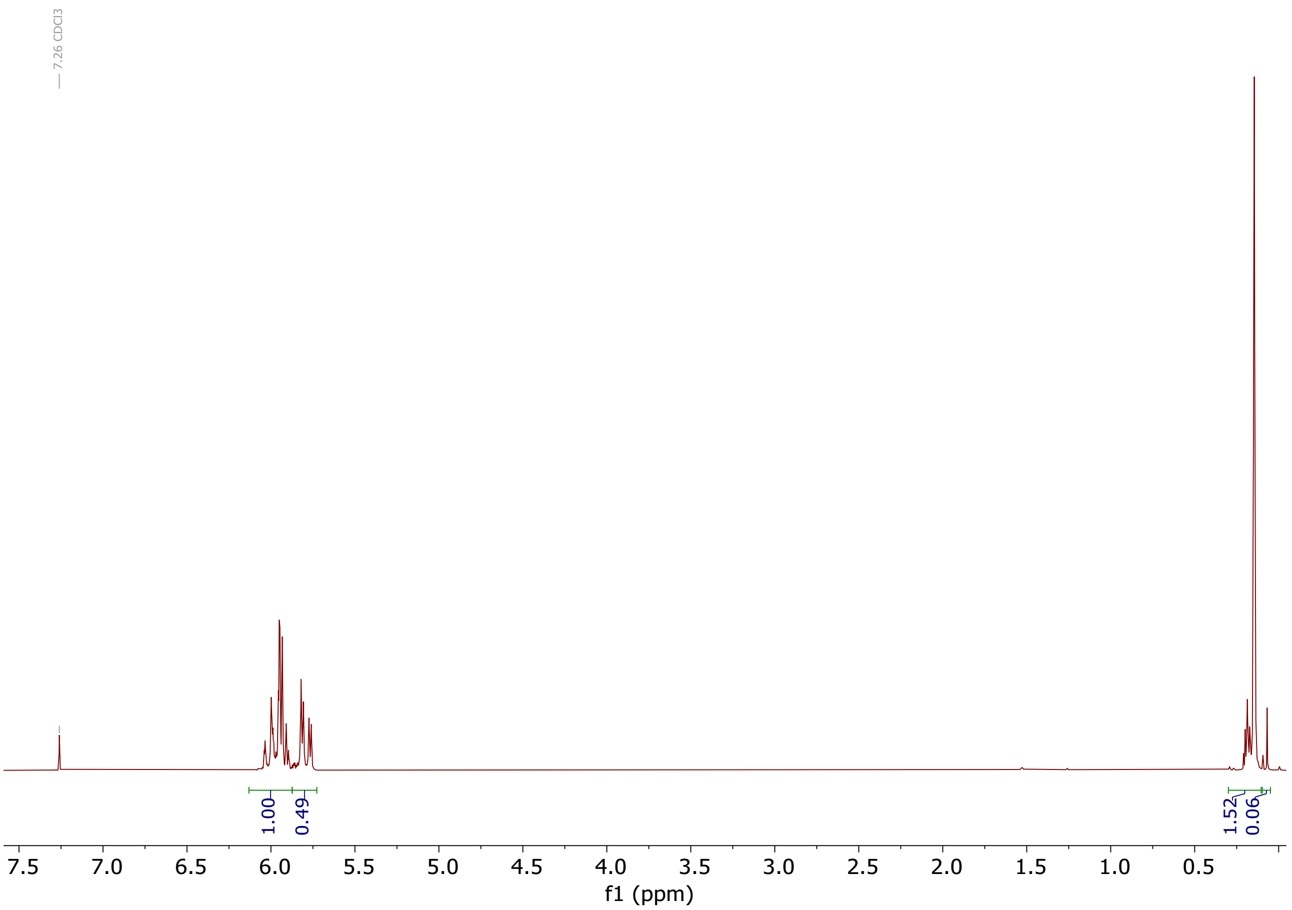

Figure S1. ^{1}H NMR of polymethylvinylsiloxane (**PSV**) in $CDCl_3$.

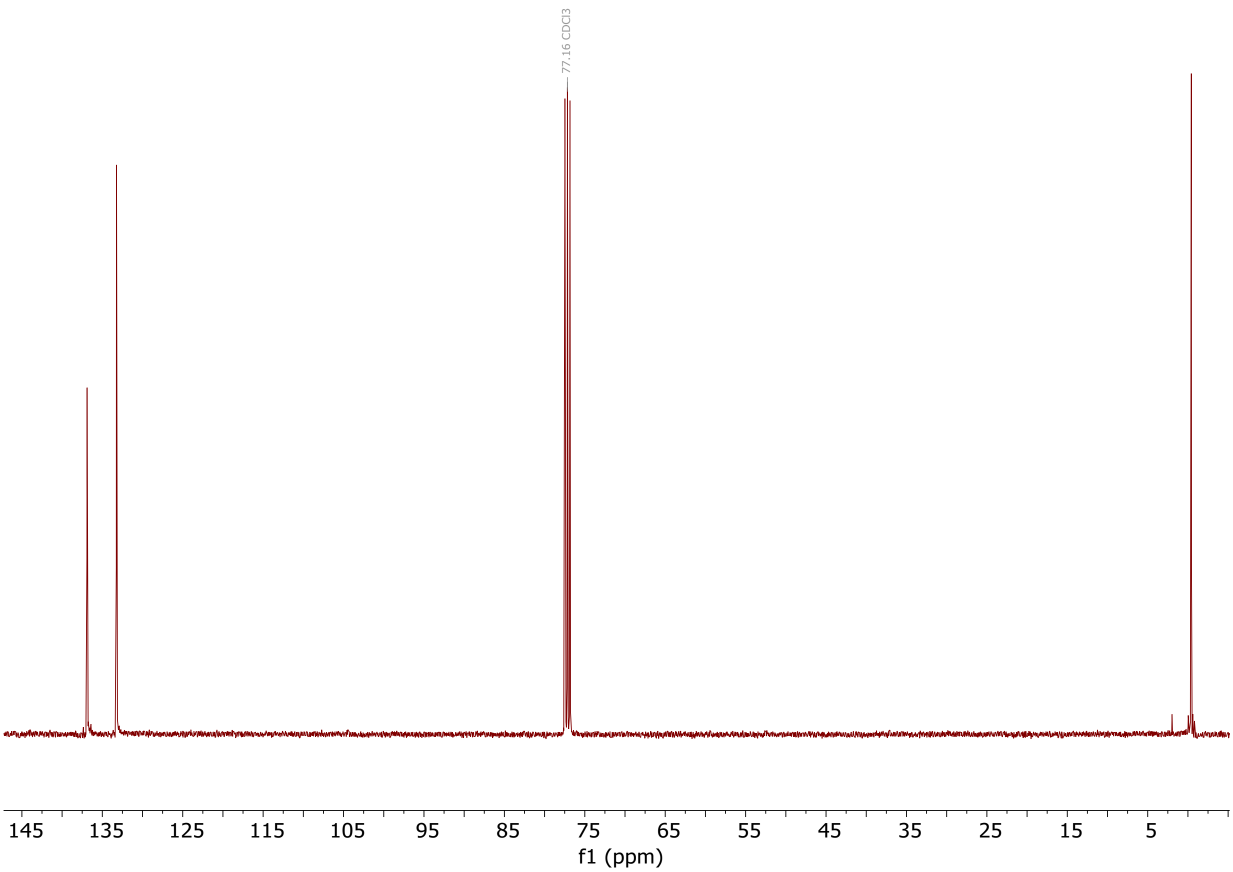

Figure S2. ^{13}C NMR of polymethylvinylsiloxane (**PSV**) in $CDCl_3$.

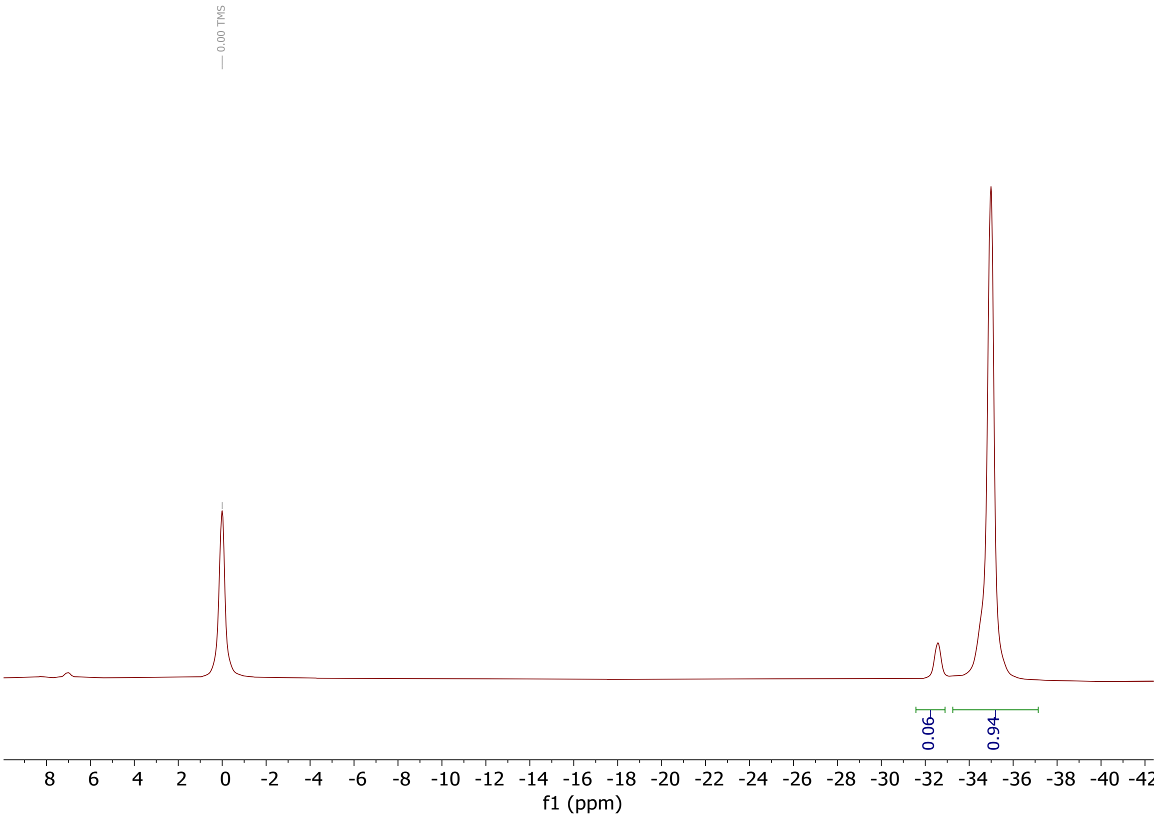

Figure S3. ^{29}Si NMR spectrum of **PSV**. The spectrum was recorded using $Cr(acac)_3$ as the relaxation agent and TMS as the reference.

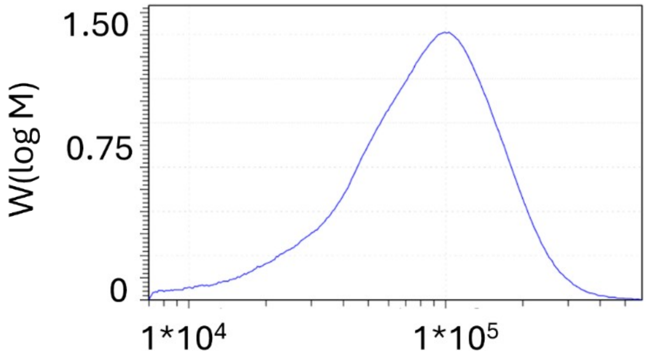

Figure S4. GPC of **PSV** using THF as a solvent and polystyrene standards (M_n = 5.98×10^4 g mol^{-1}; M_w = 9.73×10^4 g mol^{-1}; M_z = 1.36×10^5 g mol^{-1}, $Đ$ = 1.62).

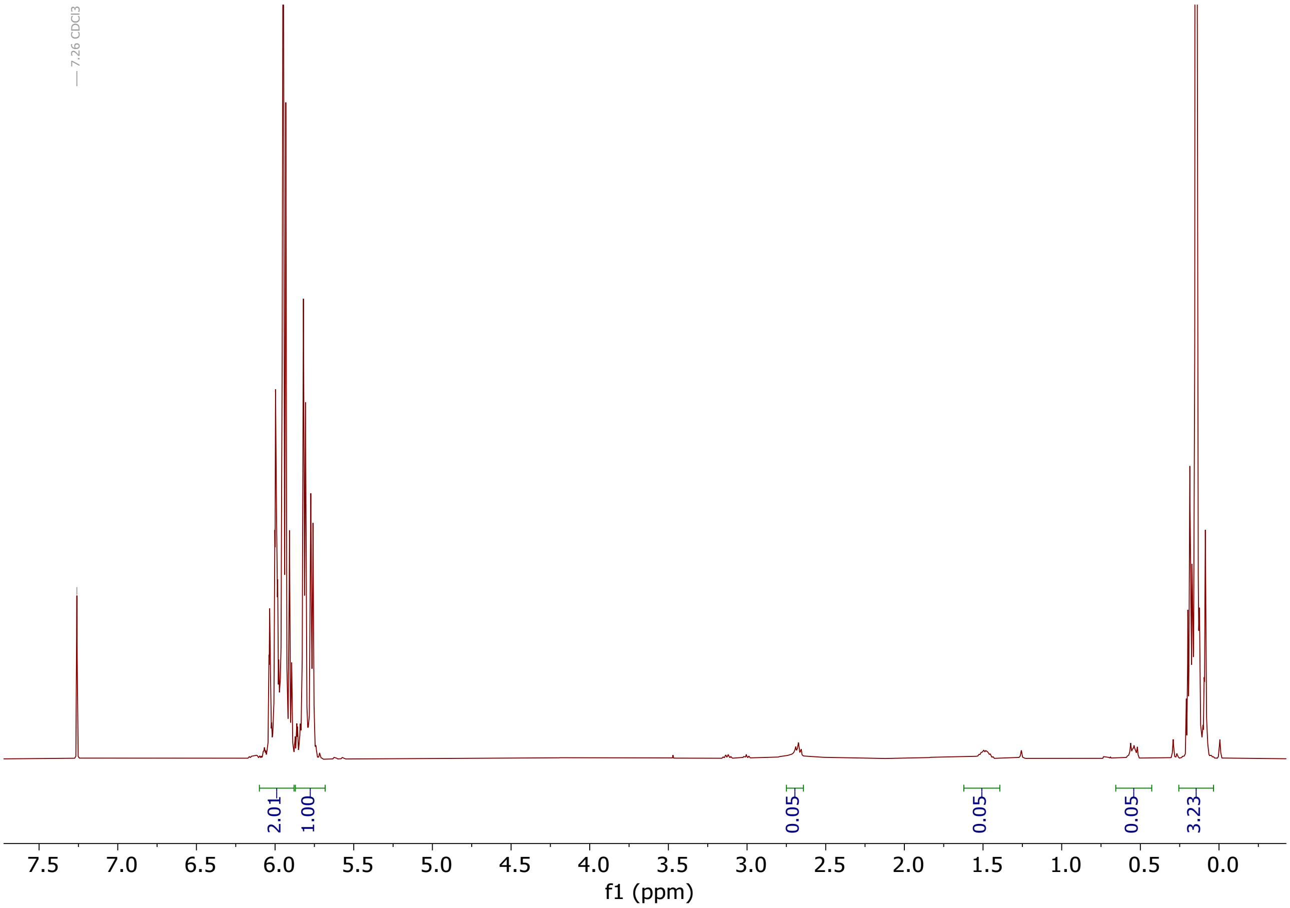

Figure S5. ^{1}H NMR of vinyl functionalized silicones with amino end functionalization (**H_2N-PVS-NH_2**) in $CDCl_3$.

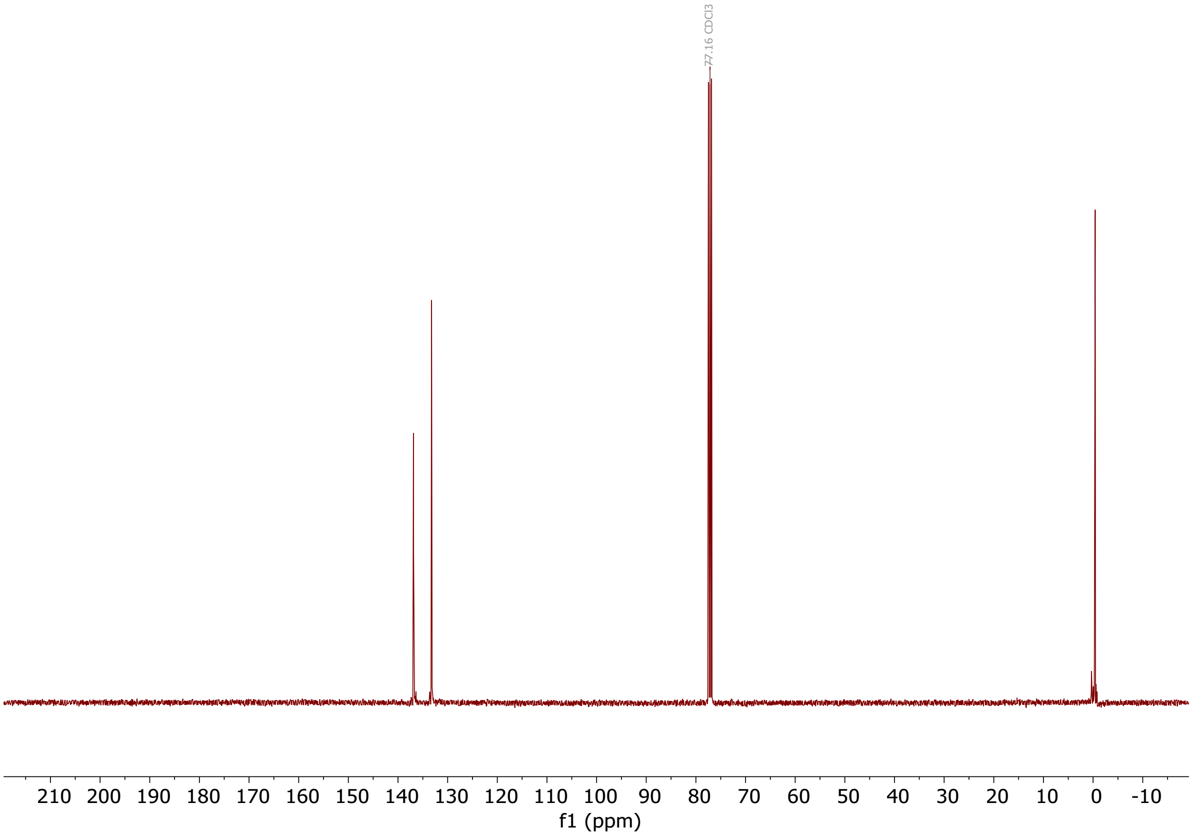

Figure S6. ^{13}C NMR of vinyl functionalized silicones with amino end functionalization (**H_2N-PVS-NH_2**) in $CDCl_3$.

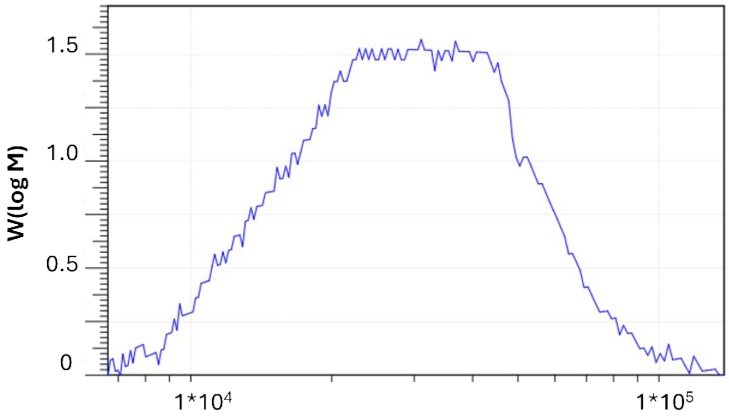

Figure S7. GPC elugram of vinyl-terminated PDMS using THF as solvent and polystyrene standards (M_n = 2.46×10^4 g mol^{-1}; M_w = 3.28×10^4 g mol^{-1}; M_z = 4.29×10^4 g mol^{-1}, $Đ$ = 1.33).

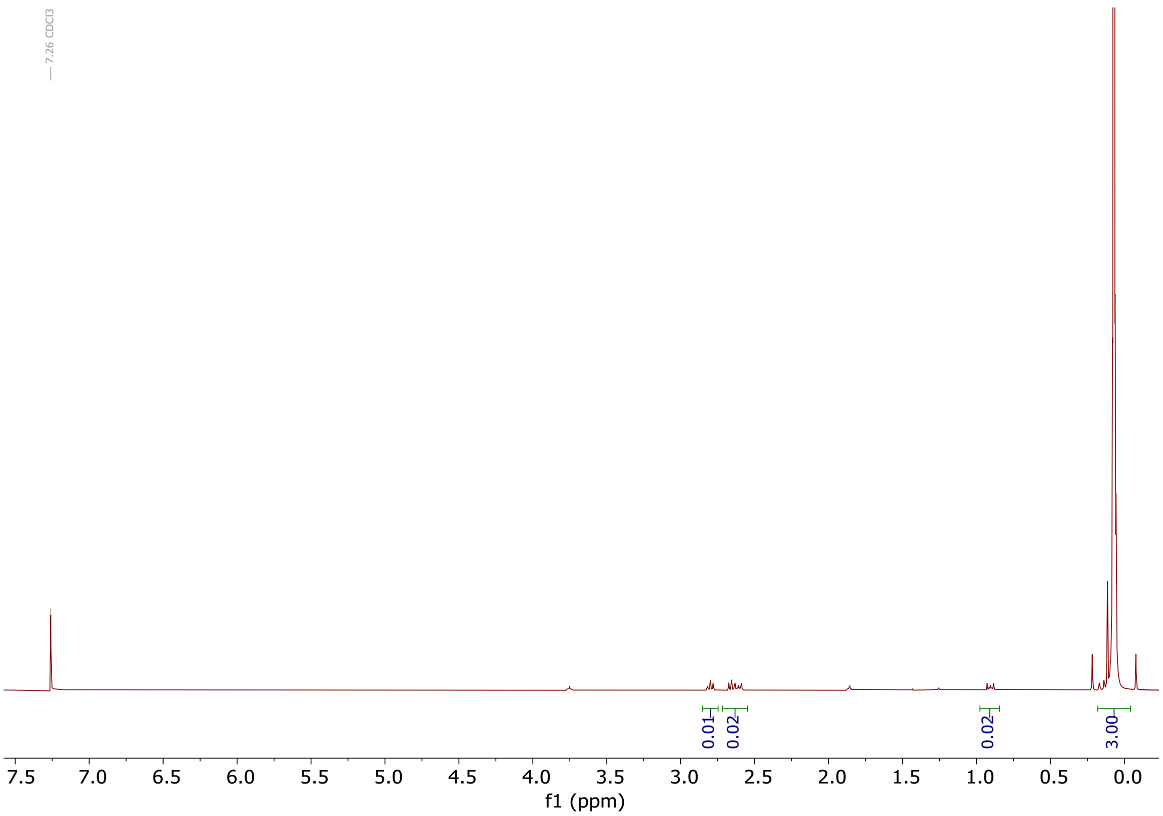

Figure S8. ^{1}H NMR of carboxylic acid end functionalized PDMS **(HOOC-PDMS-COOH)** in $CDCl_3$.

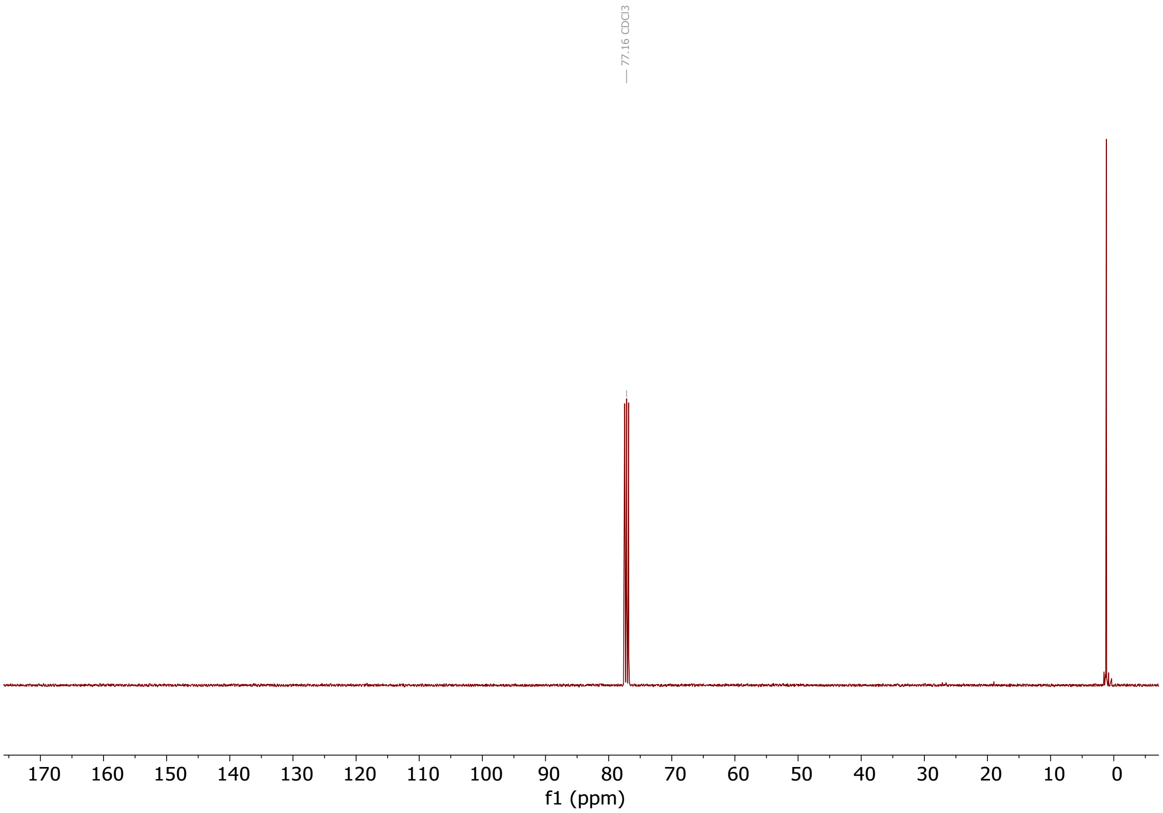

Figure S9. ^{1}H NMR of carboxylic acid end functionalized PDMS **(HOOC-PDMS-COOH)** in $CDCl_3$.

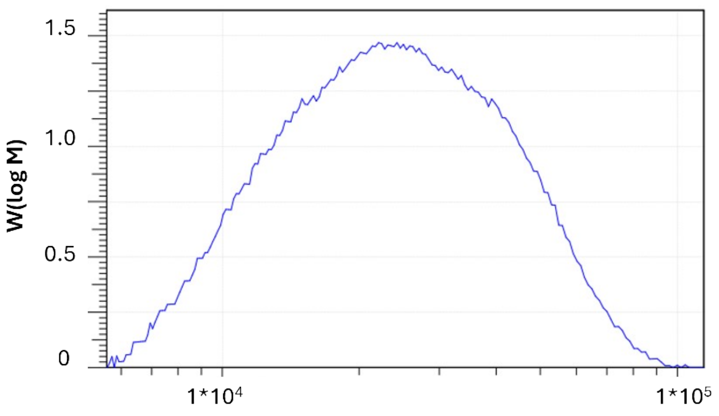

Figure S10. GPC elugram of **HOOC-PDMS-COOH** using THF as solvent and polystyrene standards (M_n = 2.00×10^4 g mol^{-1}; M_w = 2.71×10^4 g mol^{-1}; M_z = 3.56×10^4 g mol^{-1}, $Đ$ = 1.36).

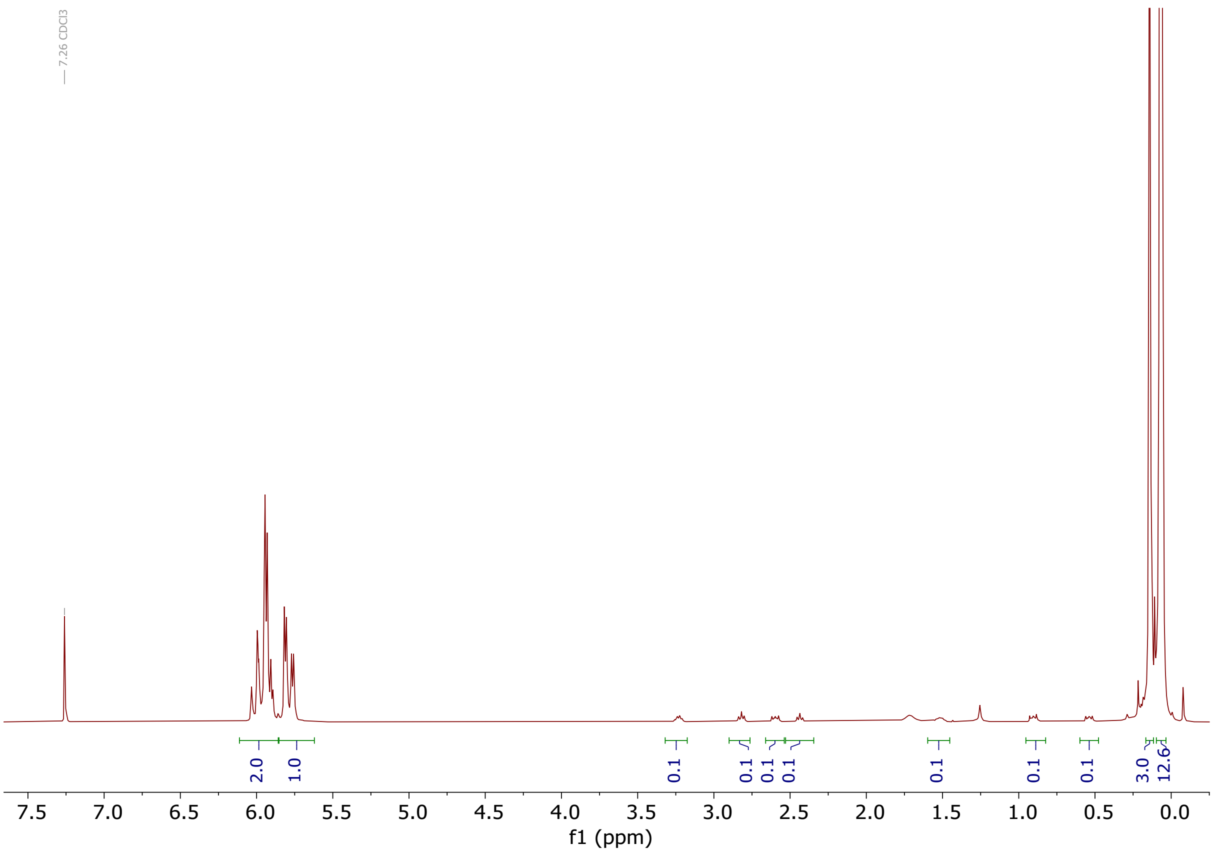

Figure S11. ^{1}H NMR of **PVS-block-PDMS** in $CDCl_3$.

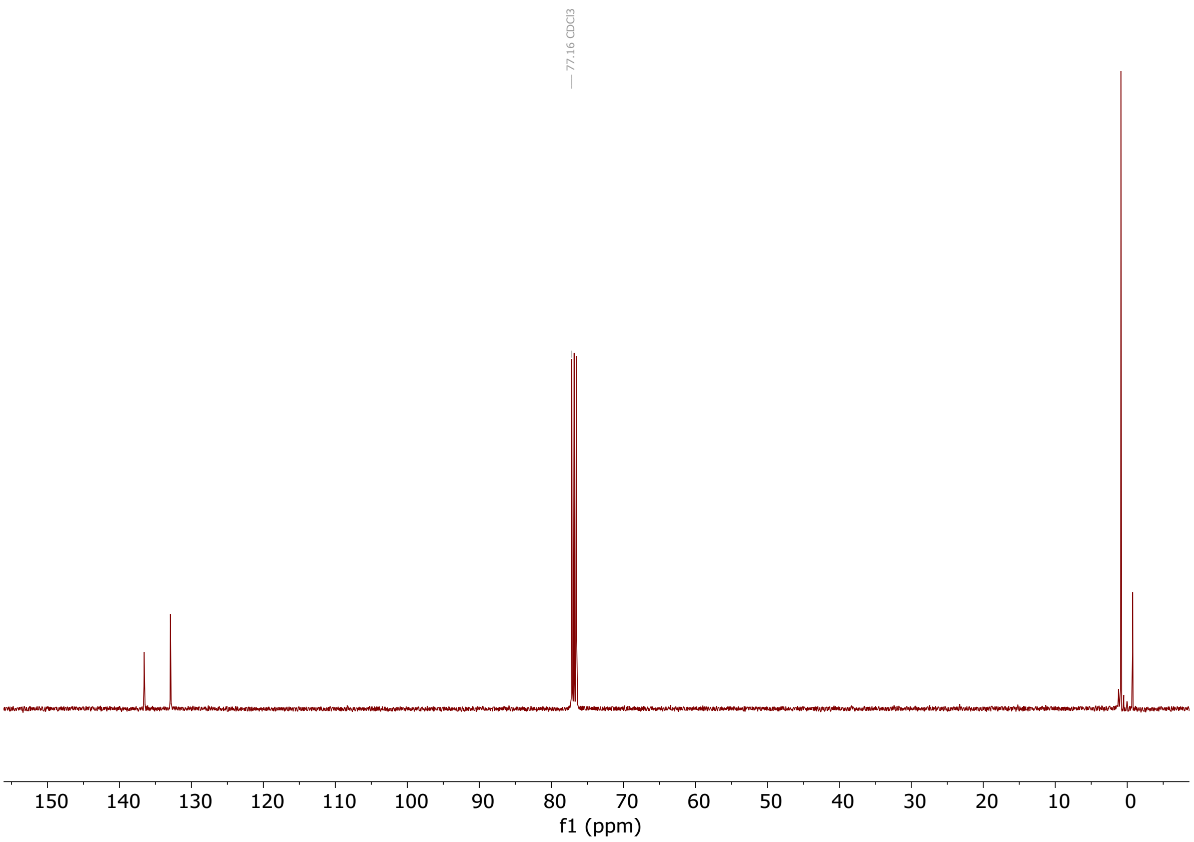

Figure S12. ^{13}C NMR of **PVS-block-PDMS** in $CDCl_3$.

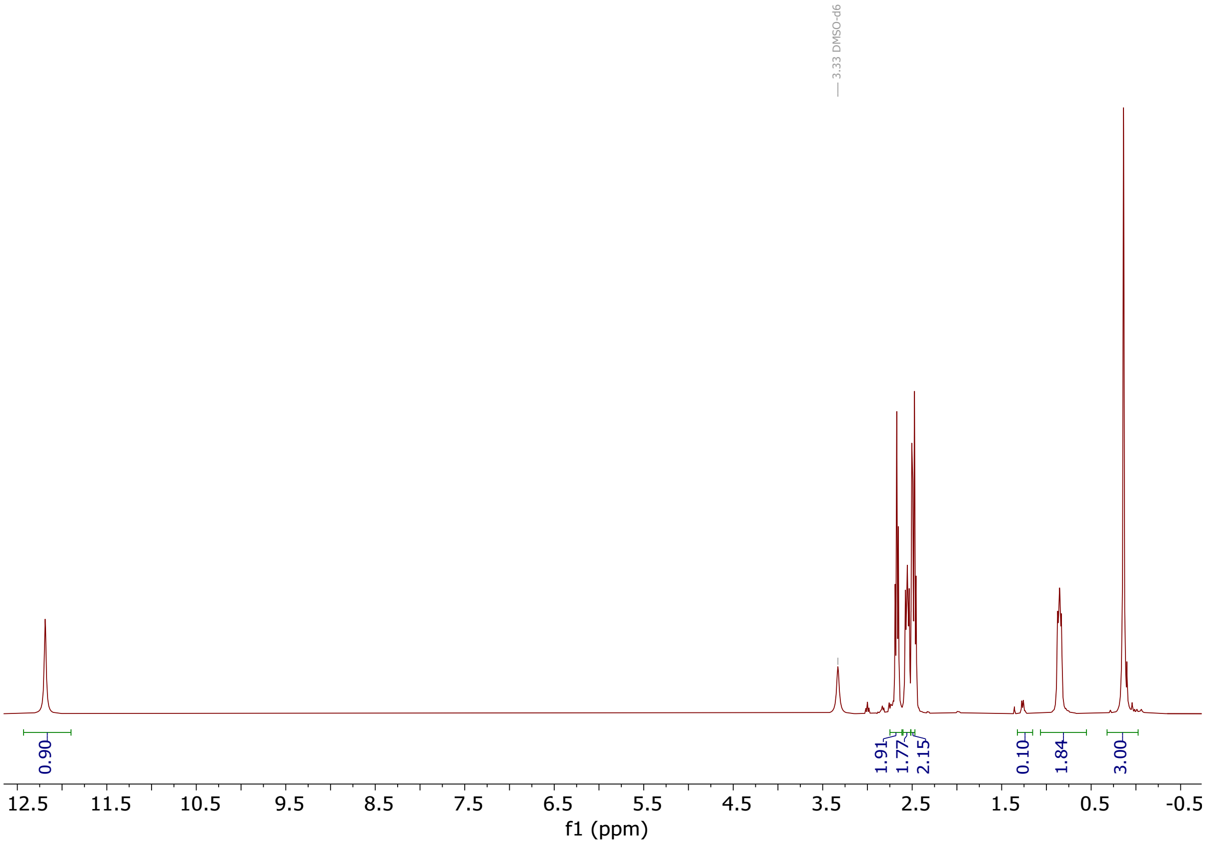

Figure S13. ^{1}H NMR of carboxylic acid functionalized silicones (**PS-COOH**) in *d*-DMSO.

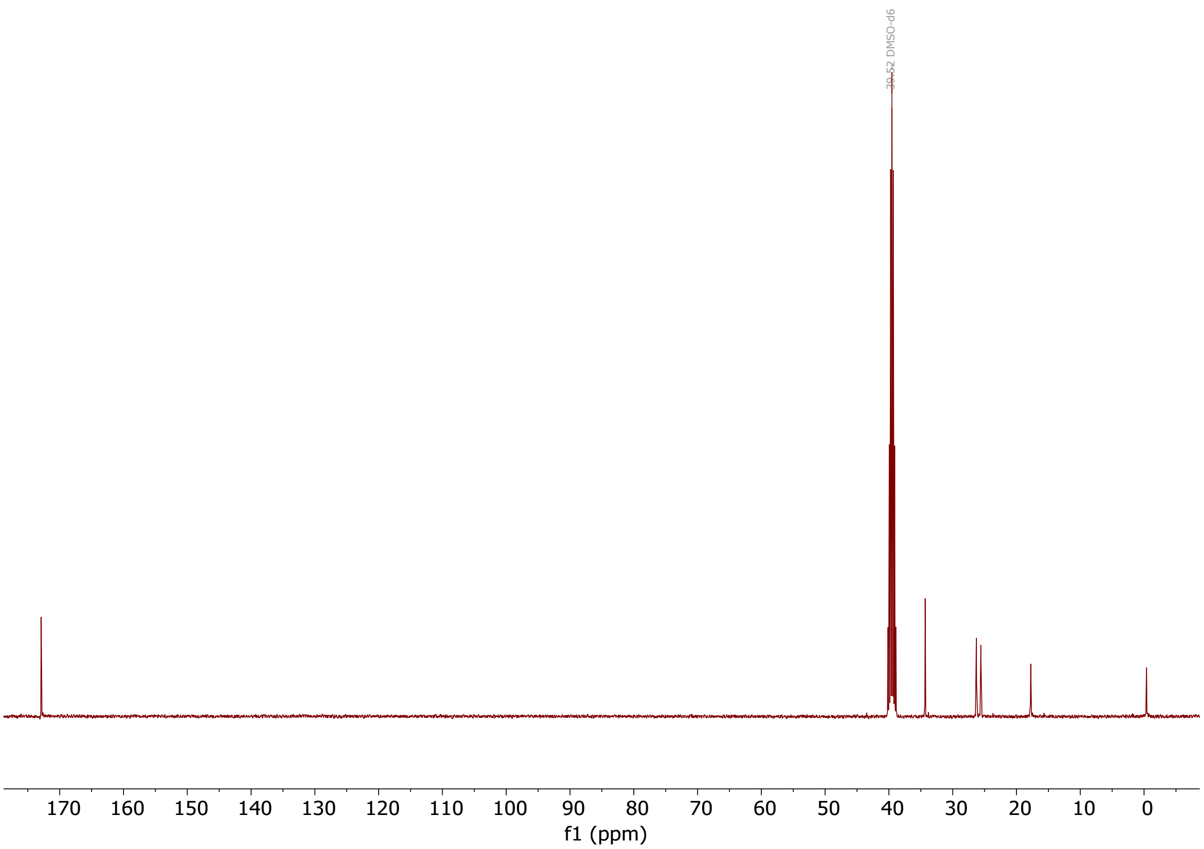

Figure S14. ^{13}C NMR of carboxylic acid functionalized silicones (**PS-COOH**) in *d*-DMSO.

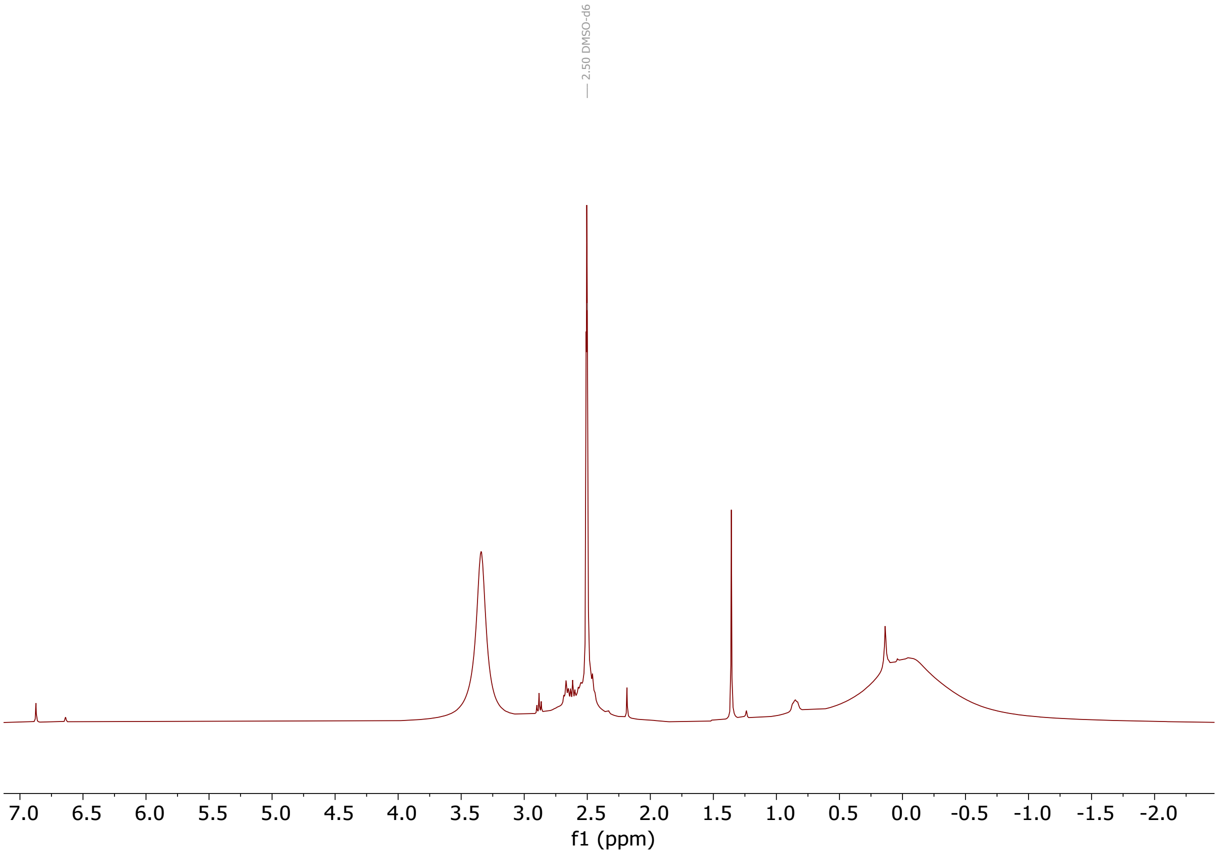

Figure S15. ^{1}H NMR of **PS-COOH-*block*-PDMS** in *d*-DMSO.

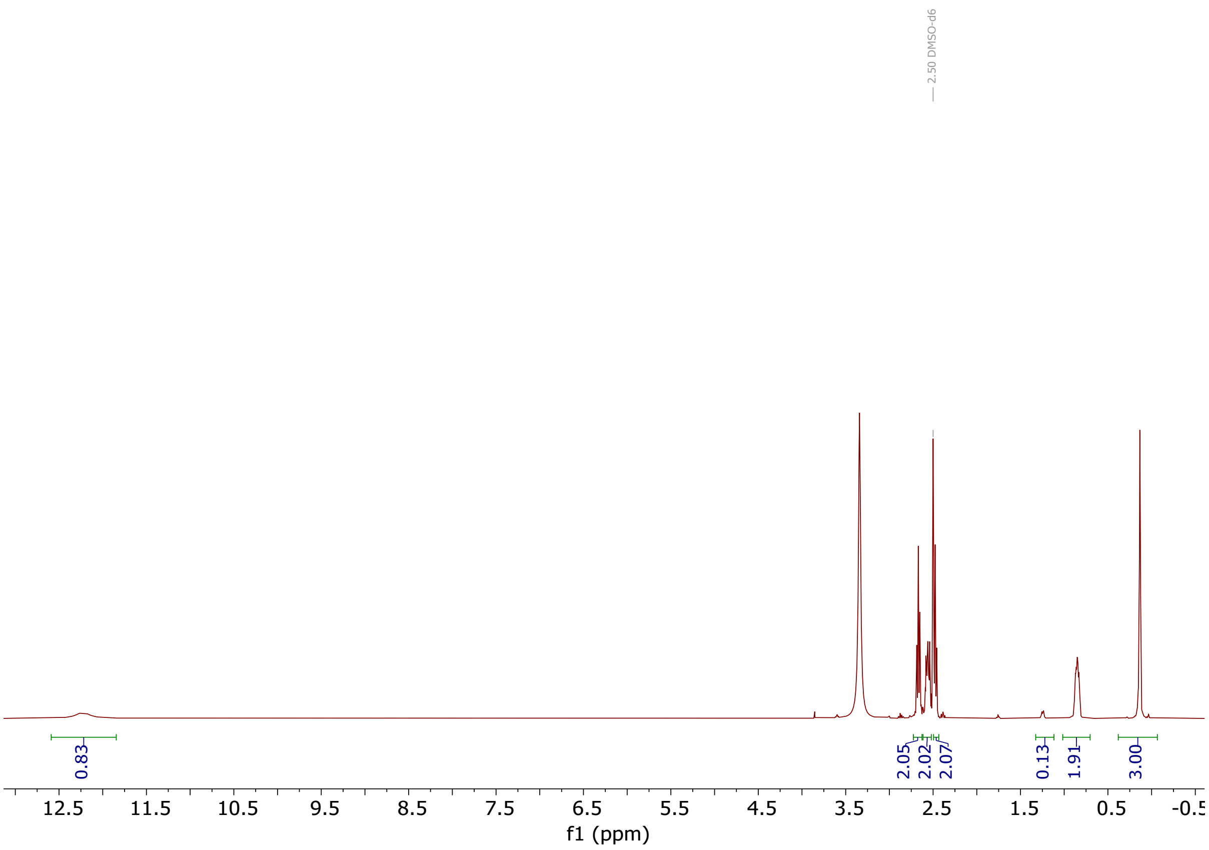

Figure S16. ^{1}H NMR of **Cycle-COOH** in *d*-DMSO.

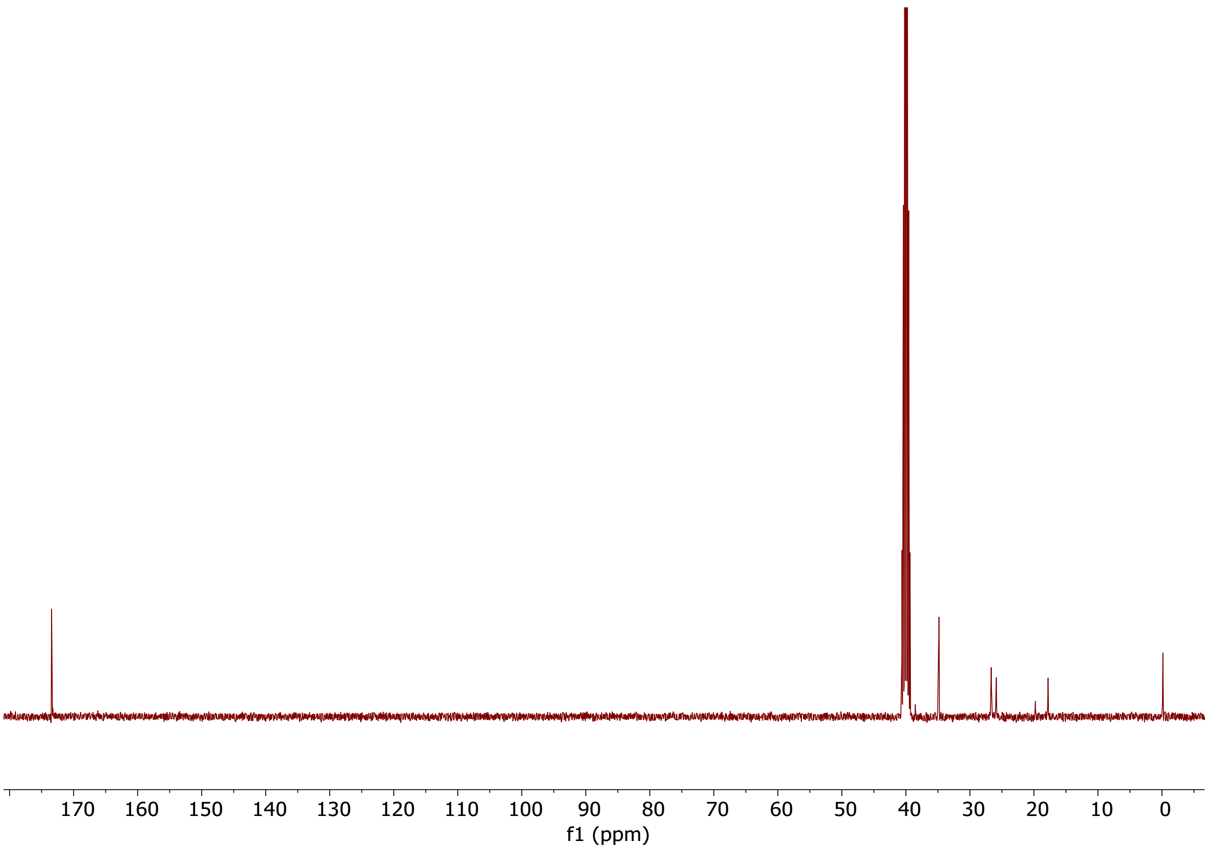

Figure S17. ^{13}C NMR of **Cycle-COOH** in *d*-DMSO.

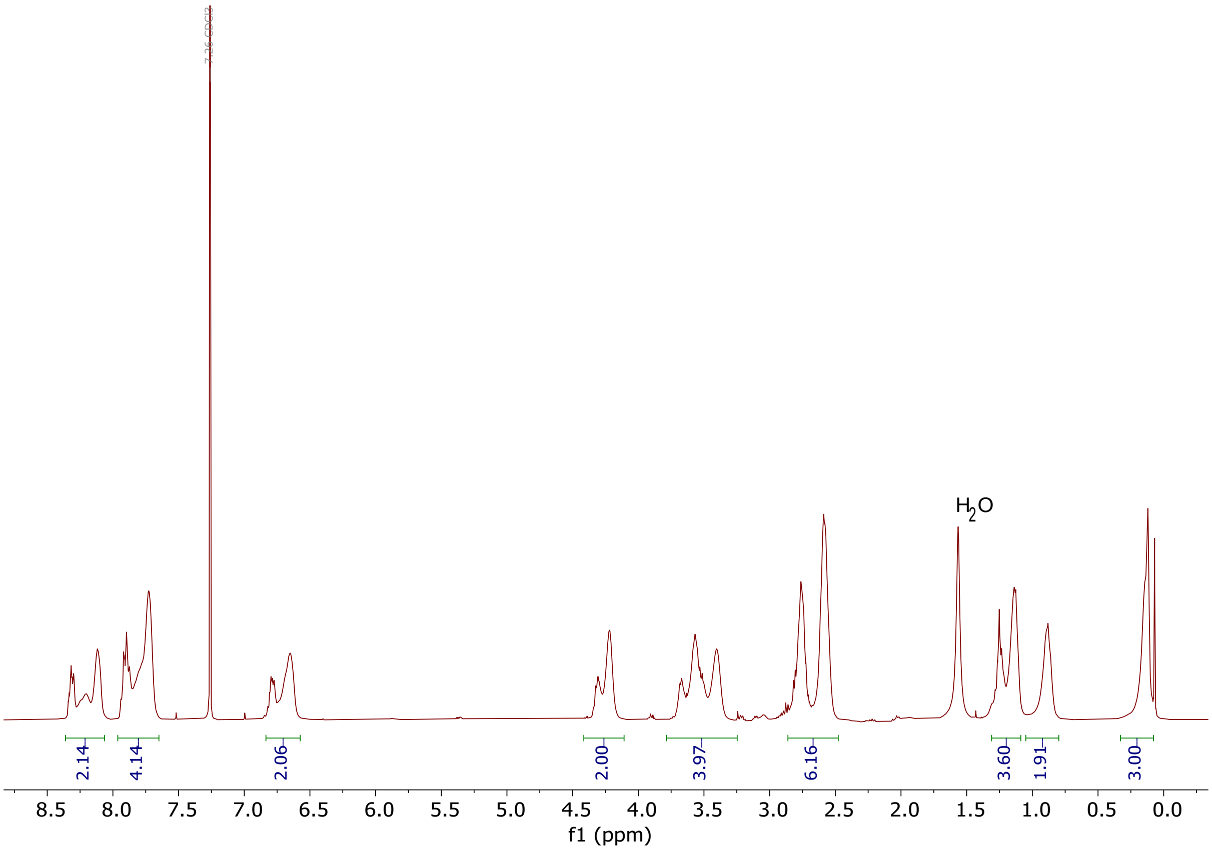

Figure S18. ^1H NMR of DR1 functionalized silicone (**homo-DR1**) in $CDCl_3$.

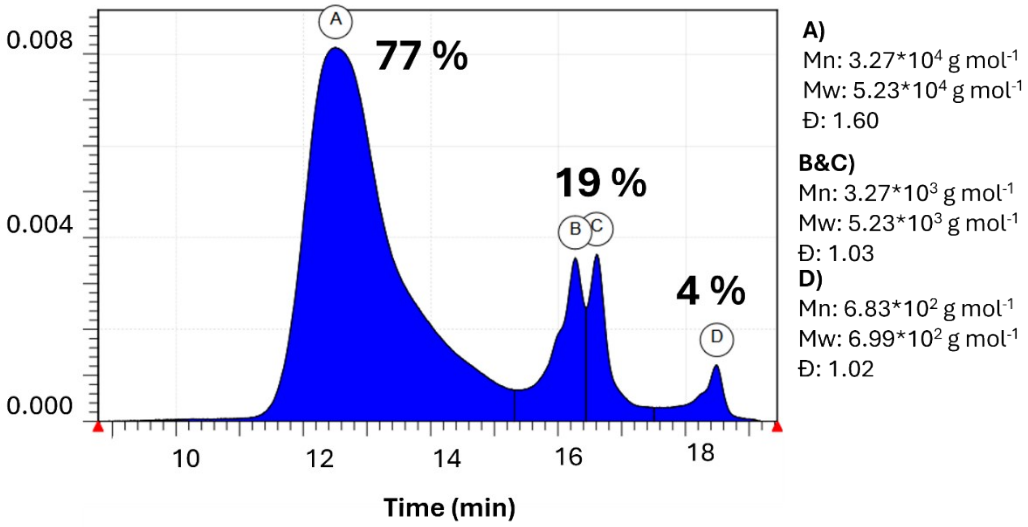

Figure S19. GPC analysis of sample **homo-DR1.**

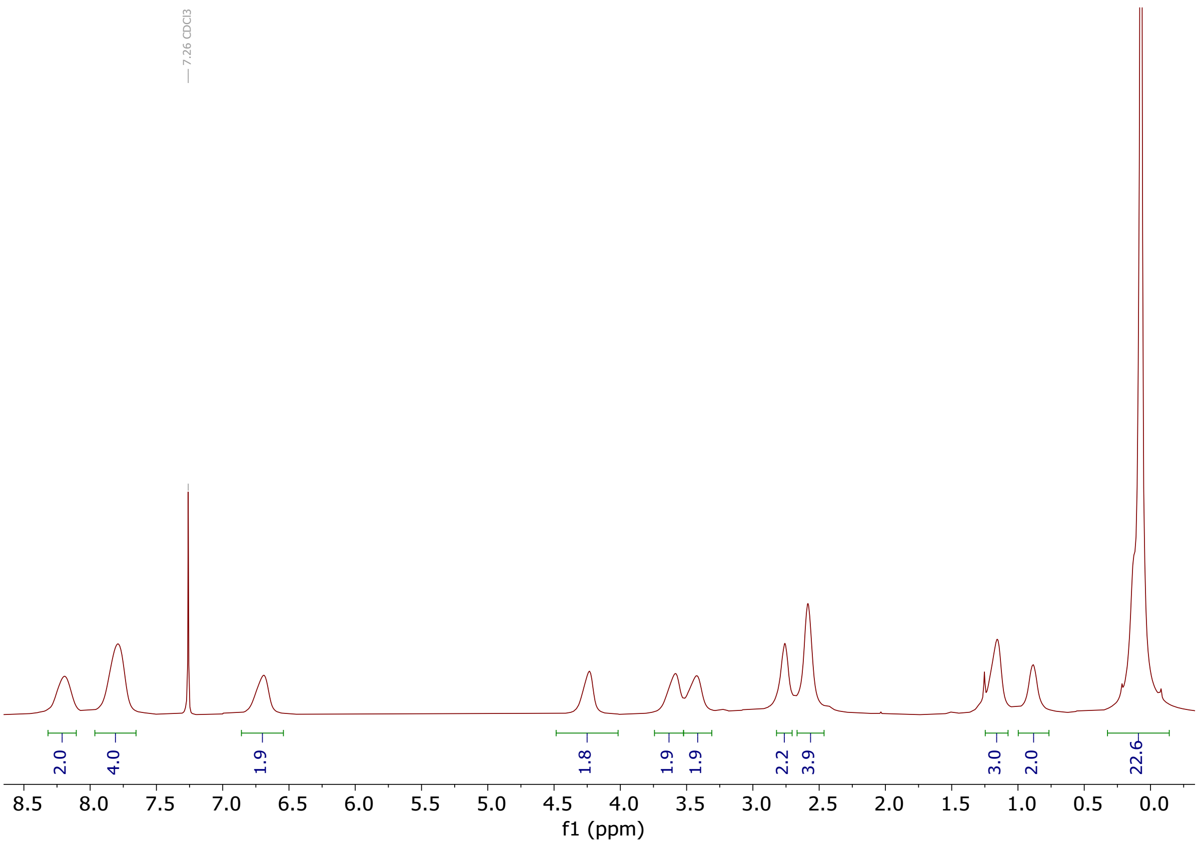

Figure S20. ^{1}H NMR of **block-DR1** in $CDCl_3$.

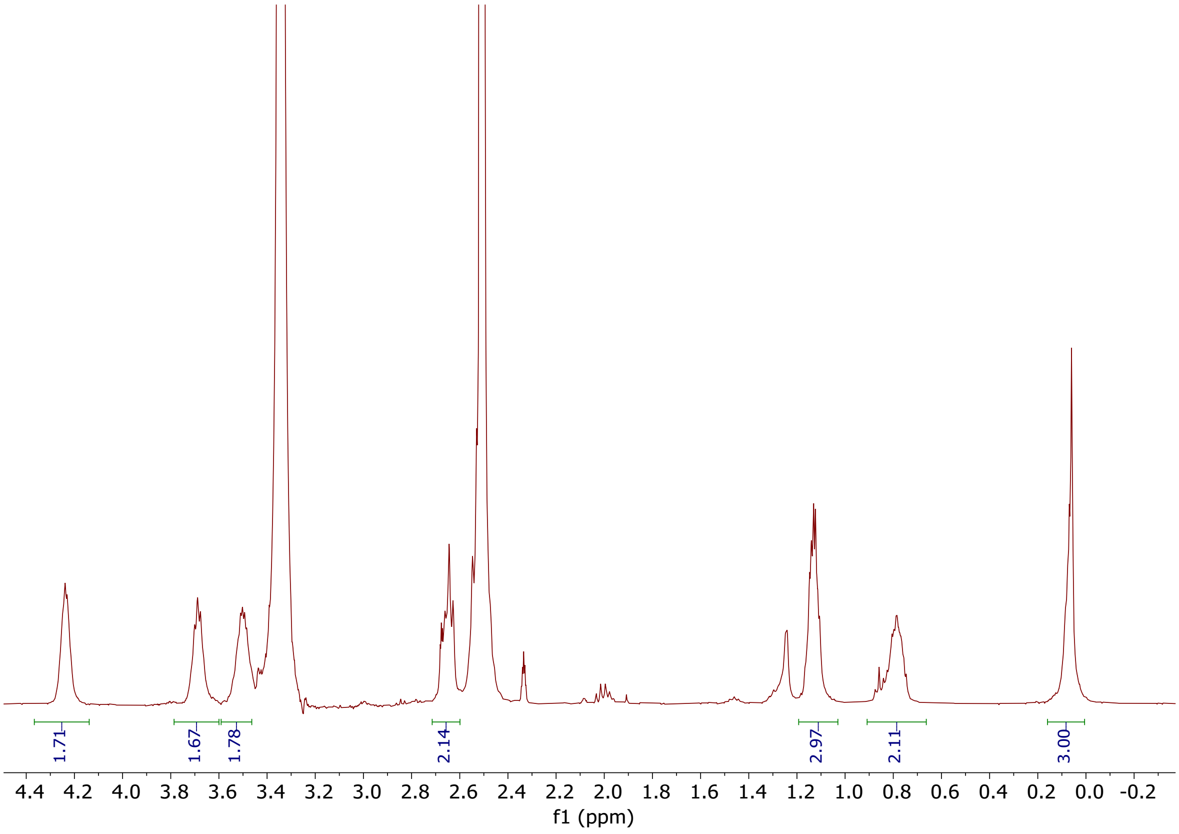

Figure S21. ^{1}H NMR of **cycle-DR1** in *d*-DMSO.

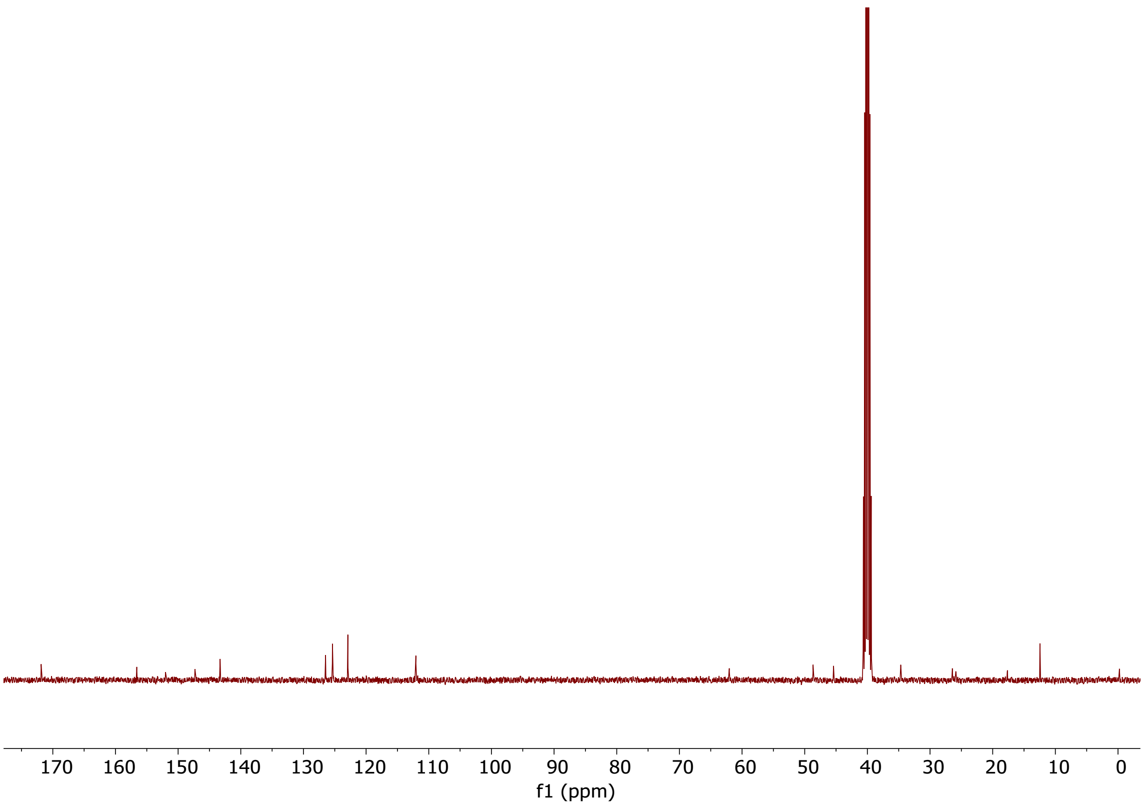

Figure S22. ^{13}C NMR of **cycle-DR1** in *d*-DMSO.

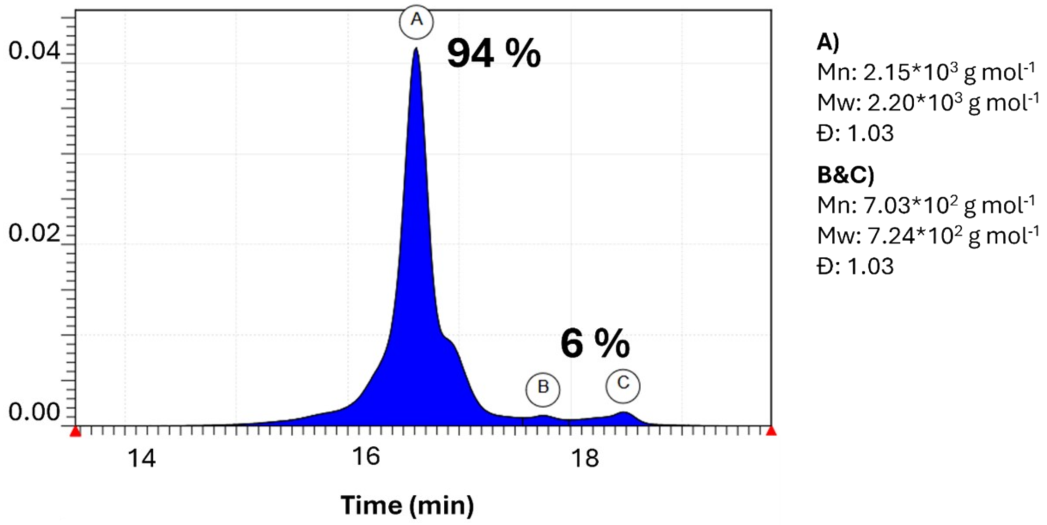

Figure S23. GPC analysis of sample **cycle-DR1.**

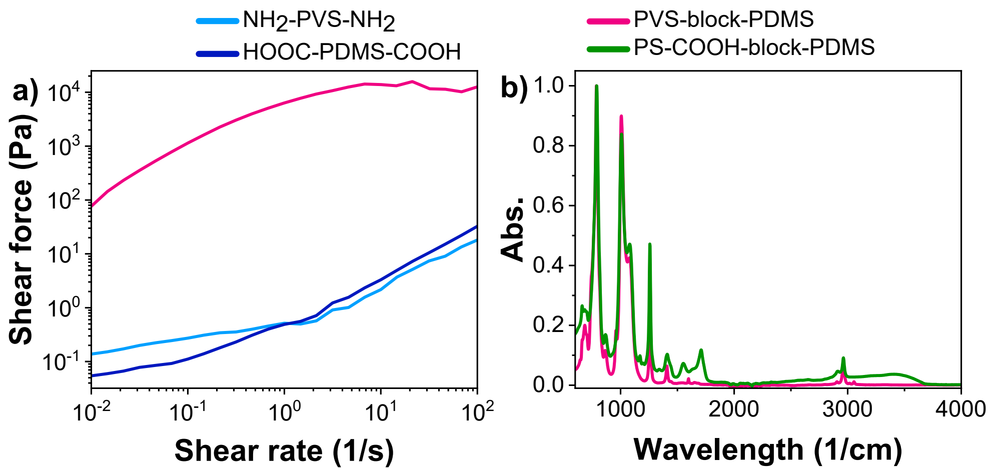

Figure S24. a) Rheological measurement of the macro monomers **NH_2-PVS-NH_2** and **HOOC-PDMS-COOH** and their reaction product **PVS-block-PDMS**. b) FTIR spectra of the vinyl functionalized block copolymer **PVS-block-PDMS** and the reaction product **PS-COOH-block-PDMS**.

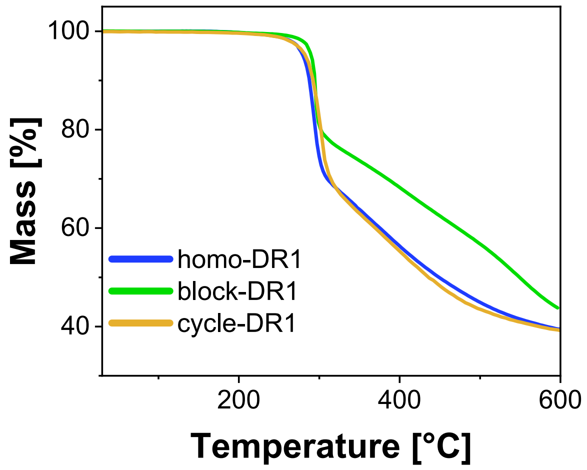

Figure S25. Thermogravimetric analysis of **homo-DR1**, **block-DR1**, and **cycle-DR1**.

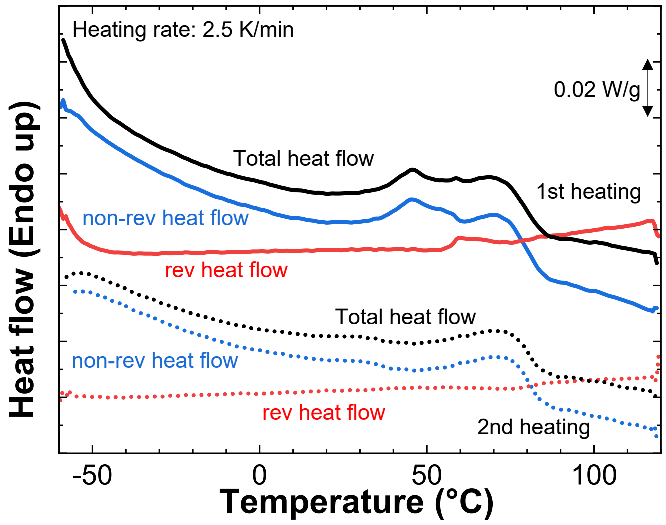

Figure S26. Modulated DSC of sample **block-DR1**: total heat flow (black); non-reversible heat flow (blue); reversible heat flow (red); first heating (straight line); second heating (dotted line)

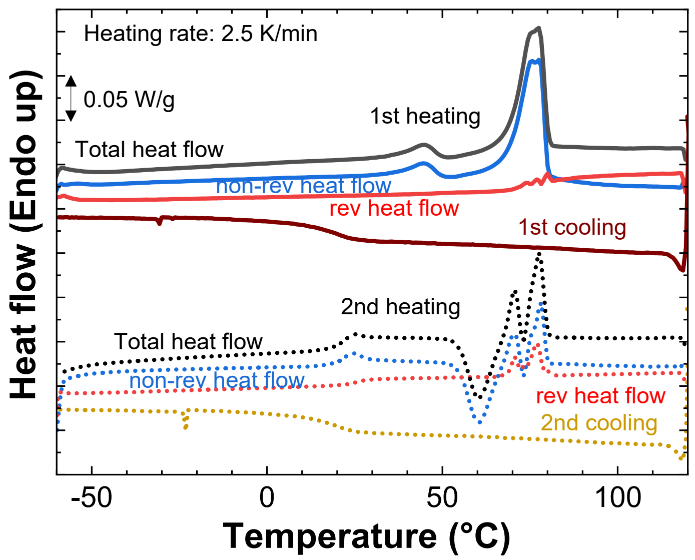

Figure S27. Modulated DSC of sample **cycle-DR1**: total heat flow (black); non-reversible heat flow (blue); reversible heat flow (red); first heating (straight line); second heating (dotted line)

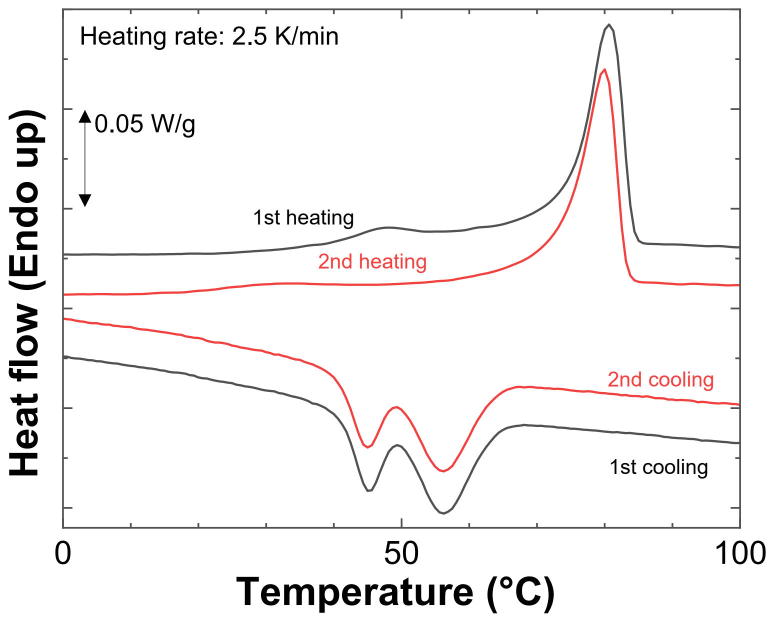

Figure S28. DSC measurement of sample **homo-DR1** at 2.5 K min^{-1}.

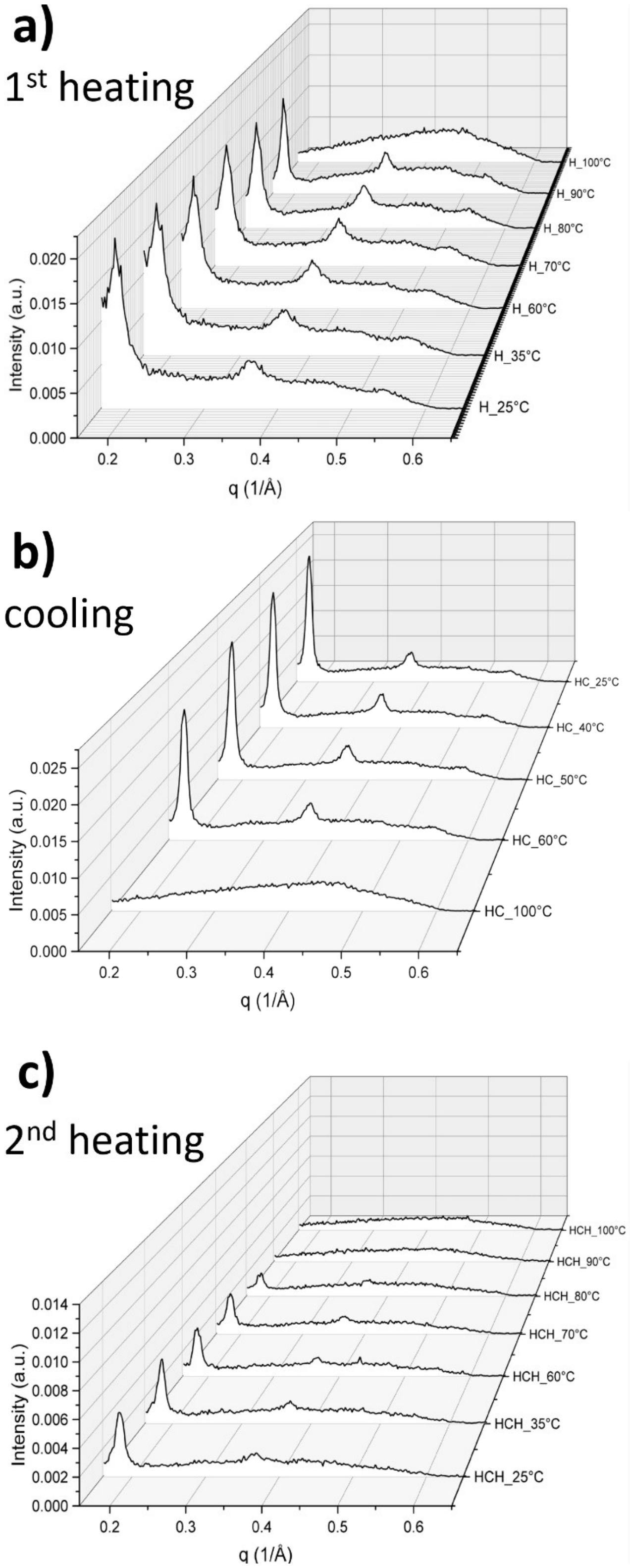

Figure S29. Temperature-dependent SAXS profiles of **homo-DR1** during heating–cooling–reheating (H–HC–HCH) cycles. a) Heating (H): SAXS patterns collected at 25, 35, 60, 70, 80, 90, and 100 °C show a clear lamellar peak series (q ≈ 0.19, 0.38, 0.57 $Å^{-1}$). b) Cooling (HC): Upon cooling from the melt, SAXS profiles recorded at 100, 60, 50, 40, and 25 °C show gradual reappearance and sharpening of the lamellar reflections. c) Second heating (HCH): Reheating at 25, 35, 60, 70, 80, 90, and 100 °C reproduces the same temperature-dependent evolution of the lamellar peak series.

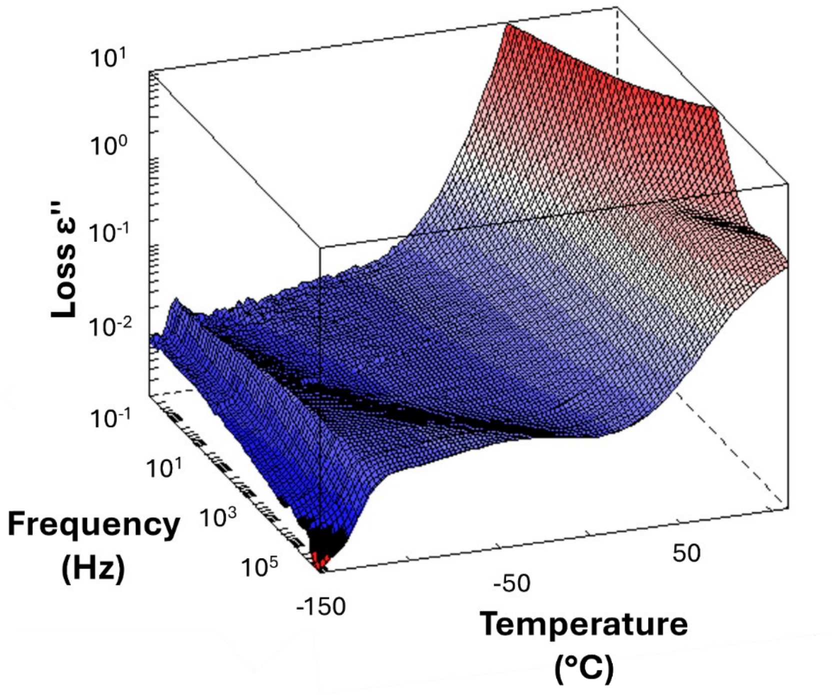

Figure S30. 3D loss plots of **block-DR1** sample plotted as a function of frequency and temperature.

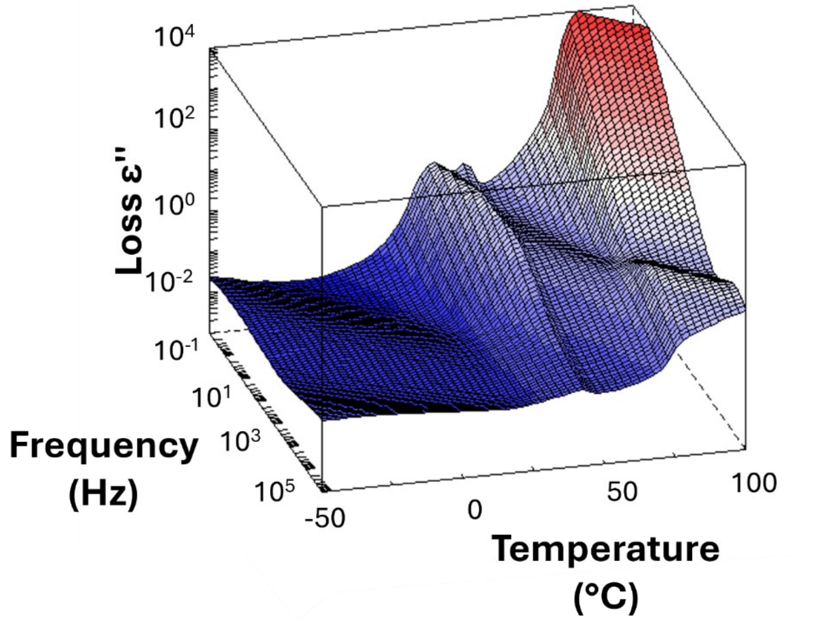

Figure S31. 3D loss plots of **cycle-DR1** sample plotted as a function of frequency and temperature.

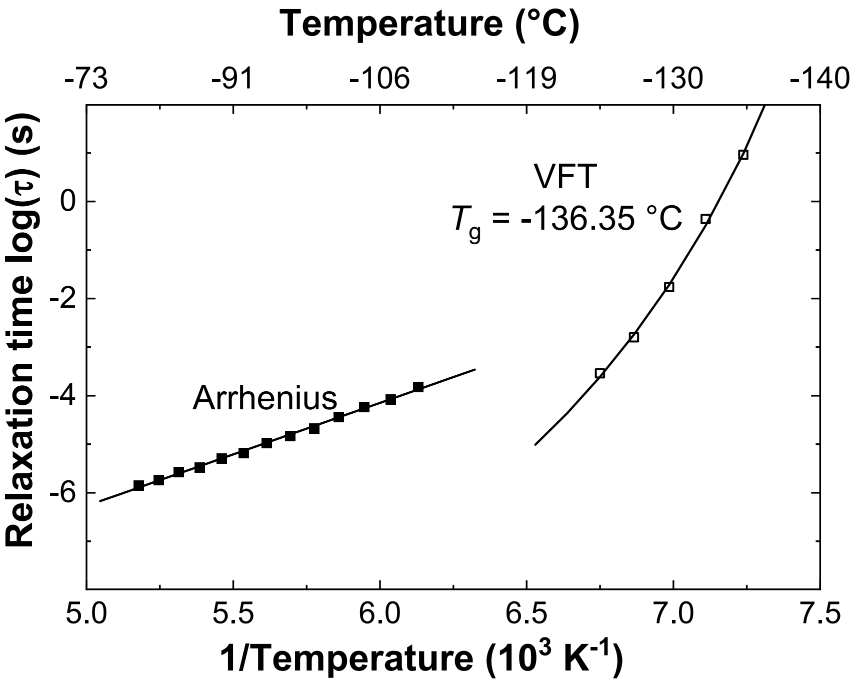

Figure S32. Arrhenius plot of the relaxation times obtained from Havriliak and Negami fits of the two relaxations observed in **block-DR1** sample.

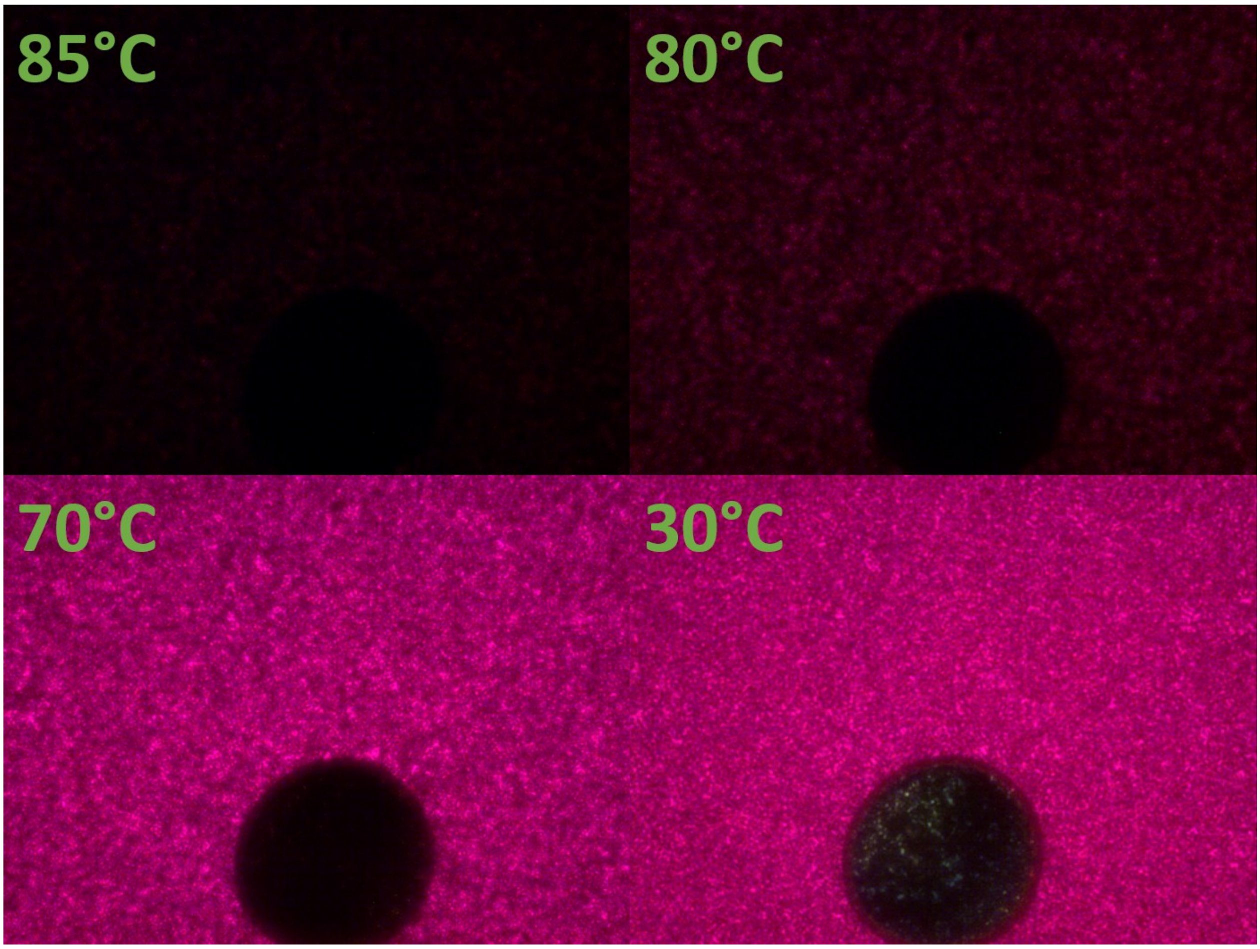

Figure S33. Temperature dependent polarized optical microscopy of **homo-DR1**.

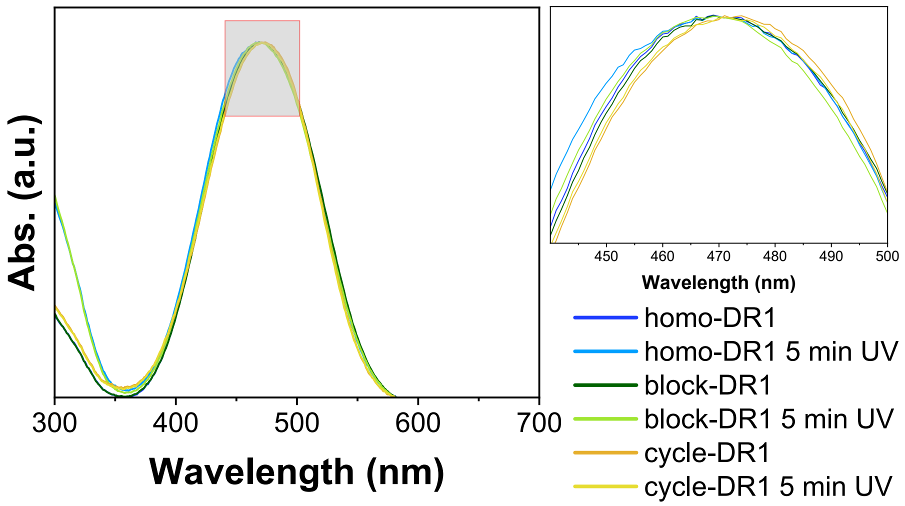

Figure S34. UV-Vis spectra of sample **homo-DR1**, **block-DR1**, **cycle-DR1** in THF before and after UV irradiation.

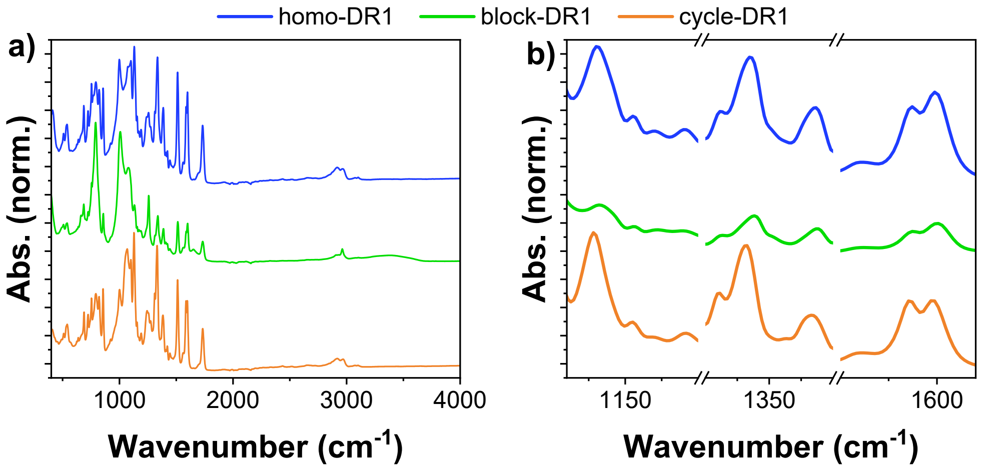

Fig. S35. a) IR-spectroscopy of sample **homo-DR1**, **cycle-DR1**, and **block-DR1**; b) highlights characteristic bands, that exhibit a shift in the wavenumbers in different samples.

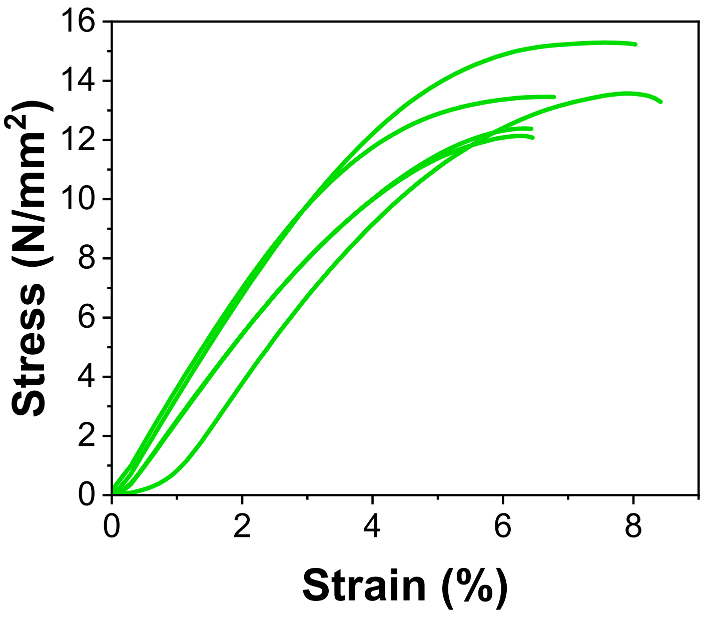

Figure S36. Tensile test of sample **block-DR1**.

Table S1. The pyroelectric response of all three samples (thickness = 100 µm) was measured under different conditions. Given is the amplitude in the current after 2000 seconds of measuring. The poling was conducted at 100°C and cooled to 20 °C under a DC field of 5 V µm^{-1}.

Poling/*Measurement* conditions	Current amplitude measured during pyroelectric measurement [A]		
	**homo-DR1**	**cycle-DR1**	**block-DR1**
No poling, *60 °C*	3.91×10^{-12} (sample 1)<br>5.16×10^{-12} (sample 2)<br>4.43×10^{-12} (sample 3)	1.04×10^{-12}	0.64×10^{-12}
No poling, *80 °C*	1.18×10^{-9} (sample 4)	0.21×10^{-9}	
No poling, *100 °C*	7.60×10^{-9} (sample 4)	0.76×10^{-9}	
No poling, *120 °C*	24.50×10^{-9} (sample 4)	1.85×10^{-9}	
Poling, *20 °C*	1.09×10^{-12} (sample 5)	0.47×10^{-12}	0.42×10^{-12}
Poling, *35 °C*	0.67×10^{-12} (sample 5)	0.96×10^{-12}	0.53×10^{-12}
Poling, *60 °C*	4.33×10^{-12} (sample 5)	0.67×10^{-12}	0.90×10^{-12}

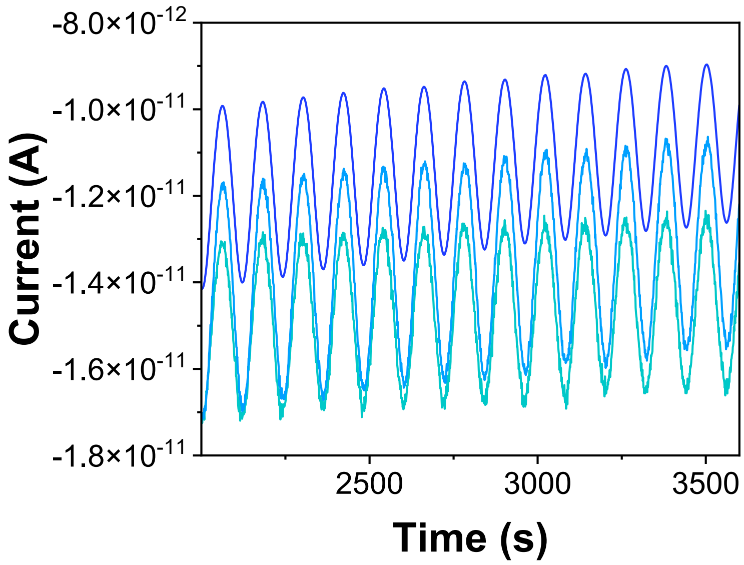

Figure S37. Pyroelectric measurement of three different unpoled **homo-DR1** at 60 °C.

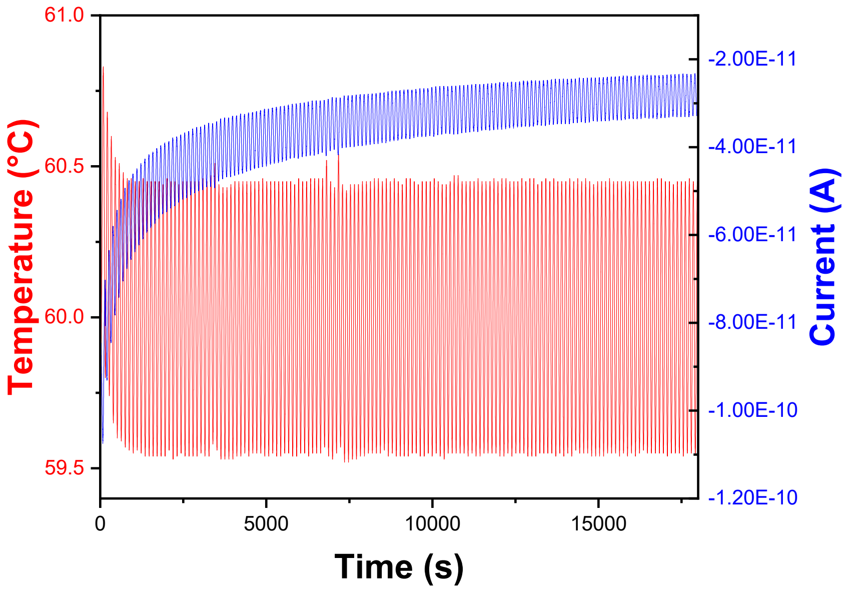

Figure S38. Pyroelectric measurement of unpoled **homo-DR1** over a period of 5 h at 60 °C.

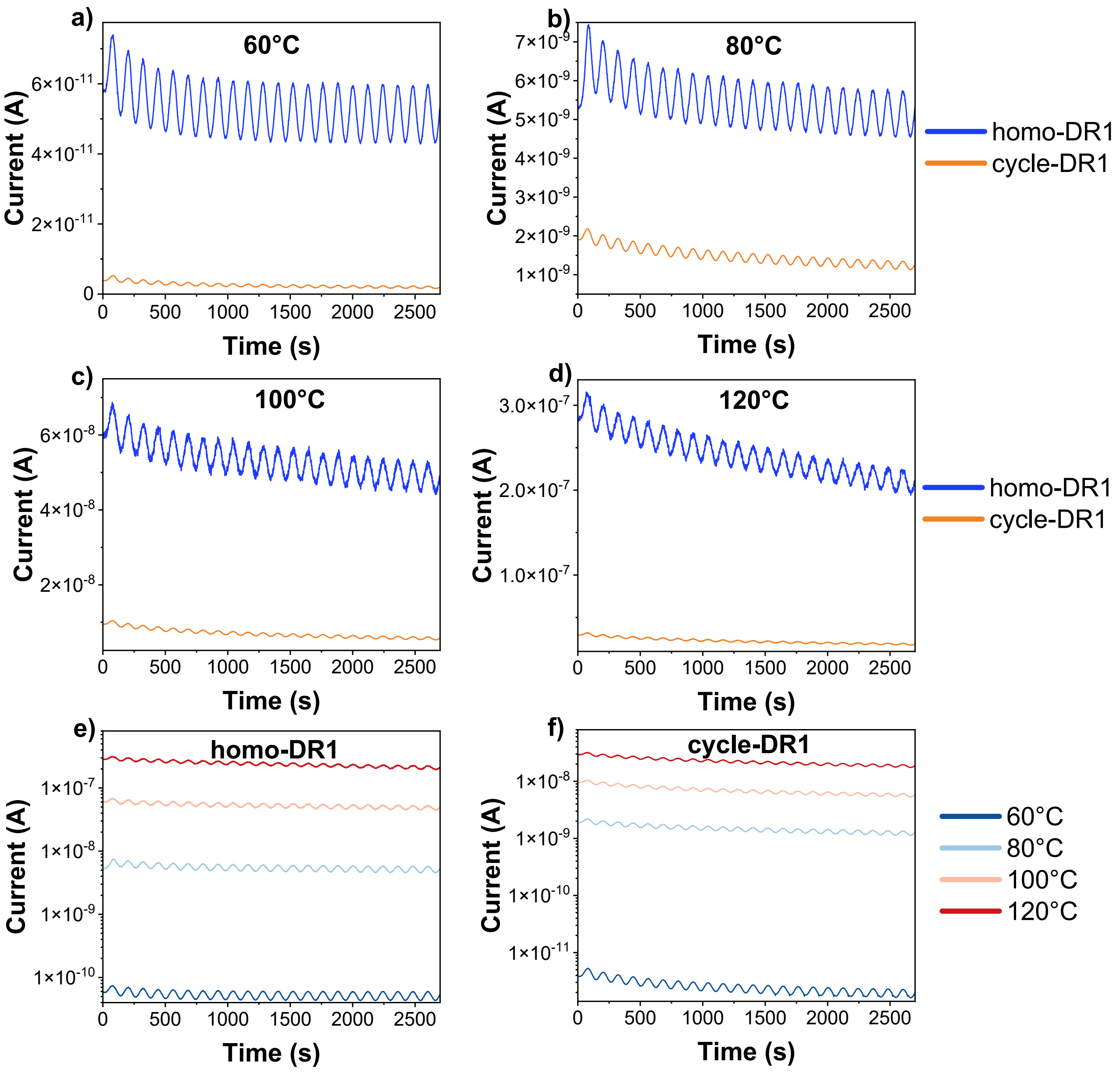

Figure S39. Comparison of the pyroelectric response of unpoled sample **homo-DR1** and **cycle-DR1** measured at 60 °C (a), 80 °C (b), 100 °C (c), and (d) 120 °C. (e) and (f) display the temperature-dependent response of sample **homo-DR1** and **cycle-DR1**.

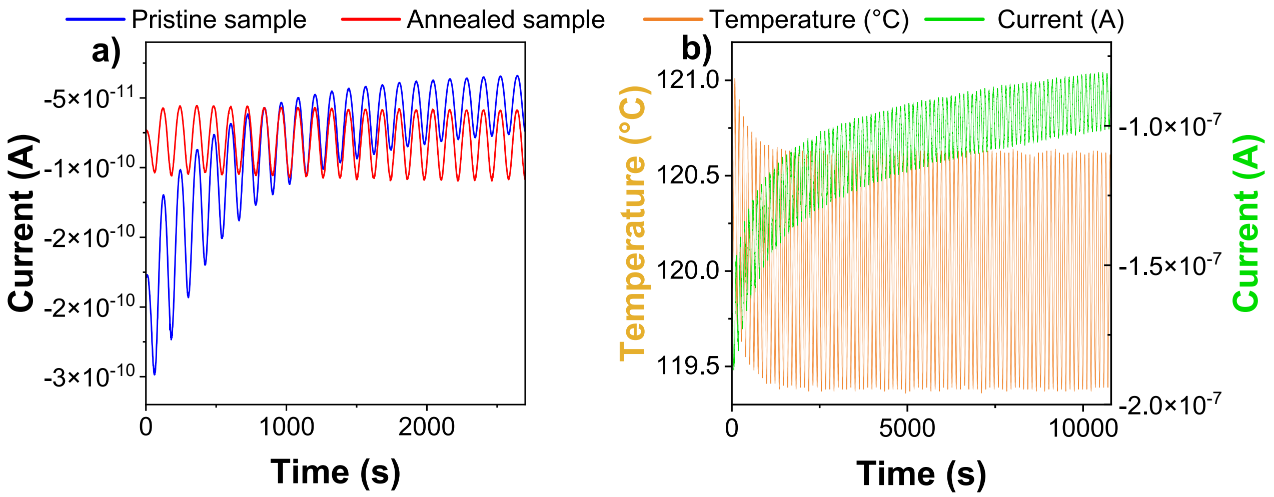

Figure S40. Comparison of the pyroelectric response of unpoled sample **homo-DR1** on interdigitated electrodes before and after annealing at 120 °C measured at 60 °C (a), stability of the pyroelectric response at 120 °C over three hours (b).
